# Chemically Cross-Linked Nanocapsules from Terpolymers of Polyethylene Glycol-Polylactide with Cationic Pendent Groups for Pgp-siRNA and Doxorubicin Codelivery in Overcoming Multidrug Resistance in Breast Cancer

**DOI:** 10.1021/acs.molpharmaceut.4c00600

**Published:** 2025-11-28

**Authors:** Zheng-Ian Lin, Tzu-Hsien Tsai, Li-Hsien Wu, Chien-Hou Lu, Chih-Yuan Hsu, Haiqing Liang, Hsin-Yu Lin, Yi-Ping Fang, Wing-Cheung Law, Cheng-Hsi Chang, Hau-En Liou, Chengbin Yang, Pei-Wen Cheng, Che-Hsin Lee, Chih-Kuang Chen

**Affiliations:** a Polymeric Biomaterials Laboratory, Department of Materials and Optoelectronic Science, 34874 National Sun Yat-Sen University, Kaohsiung 80424, Taiwan; b Department of Medical Education and Research, Kaohsiung Veterans General Hospital Kaohsiung, Kaohsiung 81362, Taiwan; c Division of Cardiology and Department of Internal Medicine, 36597 Ditmanson Medical Foundation Chiayi Christian Hospital, Chiayi 60002, Taiwan; d Department of Biological Sciences, National Sun Yat-sen University, Kaohsiung 80424, Taiwan; e Department of Fiber and Composite Materials, 34902 Feng Chia University, Taichung 40724, Taiwan; f Guangdong Key Laboratory for Biomedical Measurements and Ultrasound Imaging, School of Biomedical Engineering, Shenzhen University Medical School, 47890 Shenzhen University, Shenzhen, Guangdong 518060, P. R. China; g The Shenzhen Key Laboratory of Metabolism and Cardiovascular Homeostasis, 47890 Shenzhen University, Shenzhen 518060, China; h School of Pharmacy, College of Pharmacy, 38023 Kaohsiung Medical University, Kaohsiung 80708, Taiwan; i Department of Medical Research, Kaohsiung Medical University Hospital, Kaohsiung 80708, Taiwan; j Regenerative Medical and Cell Therapy Center, 38023 Kaohsiung Medical University, Kaohsiung 80708, Taiwan; k Department of Industrial and Systems Engineering, 26680 The Hong Kong Polytechnic University, Hung Hom, Hong Kong 999077, P. R. China; l Department of Biomedical Sciences, National Sun Yat-Sen University, Kaohsiung 80424, Taiwan; m Aerosol Science Research Center, National Sun Yat-sen University, Kaohsiung 80424, Taiwan; n College of Semiconductor and Advanced Technology Research, National Sun Yat-sen University, Kaohsiung 80424, Taiwan; o Department of Medical Laboratory Science and Biotechnology, 38023 Kaohsiung Medical University, Kaohsiung 80708, Taiwan; p Department of Medical Research, China Medical University Hospital, China Medical University, Taichung 404327, Taiwan

**Keywords:** cancer, multidrug resistance, polylactide, nanocapsules, drug/gene coloading

## Abstract

Cancer remains one of the leading causes of mortality worldwide. Among current treatment strategies, chemotherapy continues to play a central role; however, its therapeutic efficacy is markedly compromised by the development of multidrug resistance (MDR) in cancer cells. To address this challenge, a new triblock copolymer, poly­(ethylene glycol)-*b*-poly­(lactide)-*b*-poly­(cationic polylactide) (PEG-*b*-PLA-*b*-CPLA), was first synthesized and converted into biodegradable charged nanocapsules (BCNCs) without the use of surfactants or additives via our developed nanoemulsion interfacial cross-linking technique. With favorable biodegradability, BCNCs exhibit the ability to coload both hydrophobic drugs and hydrophilic genes. Compared with other drug/gene carriers, they feature controllable charge and surface poly­(ethylene glycol) (PEG) density, high colloidal stability, and sequential release of the loaded genes and drugs. When loaded with a hydrophobic anticancer drug, dehydrochlorination doxorubicin (Dox), and a hydrophilic P-glycoprotein (Pgp) silencing gene (Pgp-siRNA), the resulting Pgp-siRNA/Dox-coloaded BCNCs successfully demonstrated the ability to overcome MDR in MCF-7/ADR cells, due to enhanced cellular uptake, Pgp silencing efficiency, and effective endosomal/lysosomal escape of the loaded therapeutics.

## Graphical abstract

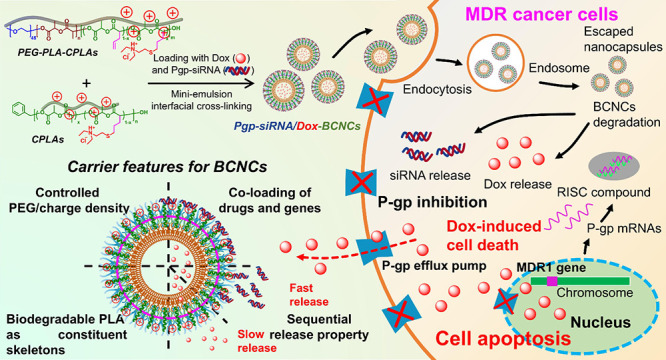

## Introduction

Cancer-related diseases have been a major cause of human mortality. In clinical practice, patients with cancer are typically treated with surgical removal of tumor tissues and radiotherapy, followed by chemotherapy.^1,2^ The success of chemotherapy has significantly improved patient survival rates. However, numerous limitations remains, such as severe adverse effects, off-target toxicity, and the development of multidrug resistance (MDR).^3^ Among these challenges, MDR is considered the most critical factor contributing to chemotherapy failure.^4–6^ The overexpression of ATP-binding cassette (ABC) transport proteins, particularly P-glycoprotein (Pgp), has been identified as a key mechanism driving MDR.^6,7^ Of various strategies to overcome MDR, gene therapy is considered one of the most effective approaches due to its ability to downregulate of Pgp expression.^4,8^ Therapeutic small interfering RNA (siRNA) can degrade Pgp-encoded mRNA by forming an RNA-induced silencing complex in the cytoplasm. Consequently, siRNA have been extensively combined with clinically used anticancer drugs, such as doxorubicin hydrochloride (Dox·HCl) and paclitaxel (Ptx) to develop siRNA/drug combinational therapies for overcoming MDR in cancer.^9,10^ Such combinational therapies have been reported to effectively improve the chemotherapeutic efficacy of anticancer drugs in MDR cancer cells both in vitro and in vivo.^11–16^ This improvement occurs because siRNA therapy can effectively inhibit Pgp-mediated drug efflux and antiapoptotic mechanism in MDR cancer cells.^9,17^ However, due to the fragile nature of siRNA, the successful delivery of siRNA into the target MDR cancer cells remains challenging. Moreover, its strong anionic charge and the relatively large molecular weight further hinder cellular uptake.^18^ Accordingly, developing an efficient gene delivery carrier is essential for the success of siRNA-based gene therapy.^19,20^ For combination therapies involving both genes and drugs, carriers must deliver hydrophilic siRNA and simultaneously load hydrophobic anticancer drugs.^21^ Such a demand to coload chemically distinct therapeutic agents considerably increases the complexity of designing and preparing of drug/gene codelivery carriers.

In this context, various types of drug/gene codelivery systems, such as liposomes,^22^ vesicles,^23^ polymeric micelles,^24^ nanoemulsions,^12^ nanobubbles,^25^ mesoporous silica nanoparticles,^26^ and other functional inorganic nanoparticles (NPs),^27^ have been developed and employed for siRNA/drug combination therapies for overcoming MDR and enhancing the antitumor efficacy of loaded anticancer drugs. Despite their promising performance in reversing MDR, several intrinsic limitations, such as poor biodegradability, limited drug loading capacity, and insufficient colloidal stability, continue to hamper their clinical availability.^28^ In particular, the ability to tune positive charge and polyethylene glycol (PEG) density is considered an important feature for optimizing MDR reversal efficacy.^29–31^ Moreover, it is ideal for the gene component of gene/drug codelivery carriers to be released first, enabling the downregulation of malignant genes in cancer cells. After sensitizing cancer cells via the delivered therapeutic genes, the encapsulated anticancer drugs can subsequently exert maximum efficacy.^32^ Among the challenges in developing gene/drug codelivery carriers, biodegradability is considered the most important factor, as it highly affects clinical applicability.^19,33^ In this context, several synthetic biodegradable aliphatic polyesters approved by the U.S. Food and Drug Administration (FDA), such as poly­(lactic-*co*-glycolic acid) (PLGA), poly­(lactic acid) (PLA), poly­(glycolic acid), and polycaprolactone, have been used as the constituent materials for gene/drug codelivery carriers.^19^ For example, Su and co-workers prepared PLGA NPs using a solvent evaporation method, followed by coating the NP surface with cationic polyethylenimine (PEI).^34^ These NPs, capable of coloading Ptx and Stat3 siRNA, have been demonstrated to effectively induce cellular apoptosis in A549 lung cancer cells. Additionally, Qiao and co-workers developed a novel copolymer PEG-*b*-PLA-Phis-ss-OEI, using oligoethylenimine (OEI), a less toxic PEI polymer, as the cationic resource.^35^ The polymer can simultaneously form a polyplex with Dox·HCl and MDR1 siRNA. This polyplex carrier effectively overcame MDR in MCF-7/ADR cells due to the efficient delivery of Dox·HCl and MDR1 siRNA. To avoid using nondegradable and toxic cationic polymers, emulsion-based methods have been employed to prepare gene/drug codelivery carriers. For instance, Paulmurugan and co-workers used a water-in-oil-in-water (W/O/W) double emulsion method to prepare PLGA-*b*-PEG NPs for the simultaneous encapsulation of a hydrophobic drug, 4-hydroxytamoxifen, and a hydrophilic miR-21 gene.^36^ This NP formulation exhibited a clear synergistic effect in reducing breast cancer cell proliferation. Similarly, Liang and co-workers utilized a double emulsion method to convert PEG-*b*-PLA into NPs.^37^ Such NPs were capable of codelivering Dox·HCl and miR-519c to HepG2 cells. By inhibition of MDR via miR-519c, this NP-mediated combination therapy successfully suppressed tumor growth in HepG2-xenografted nude mice.

Previously, our group synthesized a new biodegradable gene delivery polymer, cationic polylactide (CPLA), and successfully used CPLA-mediated siRNA therapies to suppress the proliferation of various cancer cells, including pancreatic, prostate, and leukemia cells.^27,38–42^ With its amphiphilic properties, CPLA was further converted into nanocapsules (NCs) for codelivering Dox and IL-8 siRNA in prostate cancer.^43^ These NCs demonstrated favorable biodegradability, negligible cytotoxicity, improved gene and drug loading capacity, and the ability to overcome MDR in cancer cells. However, without a stealth coating, such as a PEG layer on the surface, CPLA NCs lack sufficient colloidal stability for in vivo applications. To enhance the codelivery capabilities of PLA-based NCs, a new triblock biodegradable copolymer PEG-*b*-PLA-*b*-allyl-functional PLA (PEG-PLA-APLA) will be synthesized via ring-opening polymerization (ROP), followed by conversion of the APLA block into a CPLA block via thiol–ene click functionalization. The resulting polymers, PEG-*b*-PLA-*b*-CPLAs (PEG-PLA-CPLAs), featuring a PEG-protective layer and a controlled charge density are hypothesized to possess amphiphilic surfactant properties suitable for preparing oil-in-water (O/W) nanoemulsions, in which oil droplets are expected to be uniformly distributed in the aqueous phase with sizes typically below 500 nm due to the surfactant behavior.^44^ Moreover, PEG-PLA-CPLAs are considered to adsorb at the O/W interface. The small droplet size allows UV light to penetrate the nanoemulsions.^45^ By utilizing the remaining allyl functionalities on the CPLA block, the applied UV light can initiate radical chain polymerization of the PEG-PLA-CPLA adsorbed at the O/W interface, forming carbon–carbon cross-links. With this adsorption property and UV-initiated cross-linking capability, PEG-PLA-CPLAs can be converted into biodegradable charged NCs (BCNCs) after removal of the oil templates. During the NC preparation process, hydrophobic anticancer drugs can be loaded into the oil phase in advance, eliminating the need for toxic cationic polymers or surfactants. Following the adsorption of negatively charged Pgp-siRNA onto the positively charged surface and removal of the oil templates, Pgp-siRNA/Dox-coloaded BCNCs are expected to form.

This novel structural design endows BCNCs with several biomedical advantages. First, by using FDA-approved polymers such as PLA and PEG as constituent materials,^33,46,47^ BCNCs exhibit high clinical applicability, while PLA ensures biodegradability. Second, the presence of carbon–carbon cross-links and PEG layers is expected to improve the colloidal stability. Third, the charge and PEG density of BCNCs can be adjusted by controlling the charge density of the CPLA block and incorporating additional CPLAs as cosurfactants during the nanoemulsion interfacial cross-linking process. Fourth, the CPLA layer, which adsorbs negatively charged genes, was observed to degrade faster than the PLA core, which encapsulates hydrophobic anticancer drugs in the BCNCs. Thus, they are expected to exhibit a faster release rate of Pgp-siRNA than Dox.^47,48^ In addition, the degradable nature of the CPLA segment is anticipated to promote the unpacking efficiency of the adsorbed Pgp-siRNA. Such a sequential release profile and unpacking efficiency are hypothesized to enhance the antitumor efficacy of Dox as Pgp is downregulated in advance.

Compared with the polymeric NP-based gene/drug codelivery carriers prepared via precipitation, micellization, or double emulsion methods, BCNCs offer significant advantages. These include the elimination of nondegradable agents such as PEI, poly­(vinyl alcohol), spermidine, or cholesterol, and employing tedious preparation procedures by virtue of the unique constituent polymers and novel carrier structural design.^34–37^ In particular, relative to previously developed polymeric NPs, BCNCs are distinguished by their cross-linked structure and fast-degrading gene adsorbing polymer segment, which are expected to confer enhanced in vivo stability as well as increased gene unpacking efficiency. Based on these material features, well-defined PEG-PLA-CPLAs were first synthesized. BCNCs with optimal formulation were characterized in terms of colloidal stability, particle size, and zeta potential. Using the optimized formulation, Pgp-siRNA/Dox-coloaded BCNCs were evaluated for cellular uptake, Pgp silencing efficacy, endosomal/lysosomal escape, and MDR reversal efficacy in MCF-7 and MCF-7/ADR cancer cells. In addition, their MDR reversal capability was demonstrated in another MDR cancer cell line, MES-SA/Dx5. Ultimately, a preliminary in vivo study was conducted to evaluate the antitumor efficacy and biosafety of Pgp-siRNA/Dox-coloaded BCNCs.

## Experimental Section

### Measurements

Nuclear magnetic resonance (^1^H NMR) spectra of polymer samples were recorded on a Varian INOVA 500 500 MHz spectrometer at 25 °C, using CDCl_3_ as the solvent. Gel permeation chromatography (GPC) analyses were performed on a JASCO PU-4180 Plus system equipped with an RI-4030 detector. *N*,*N*-Dimethylformamide (DMF) was employed as the eluent, and the chromatographic column contained cross-linked divinylbenzene particles with a particle size of 5 μm and a pore size of 103 Å. Calibration curves to determine the number-average molecular weight (*M*_n_) of test samples were based on polystyrene standards. Polydispersity index (PDI) values of test samples were acquired from the used software. Fourier-transform infrared spectroscopy (FTIR) spectra were measured on an IR spectrometer (Nicolet iS50/Thermo Fisher Scientific) at a scanning rate of 500 to 4000 cm^–1^ with an increment of 1 cm^–1^ for 32 scans via an attenuated total reflectance sampling device. A dynamic light scattering (DLS) (nano-ZS90/Malvern, Inc.) instrument was employed to obtain the intensity-average hydrodynamic diameters of BCNC-relevant samples in an aqueous solution. Measurements were conducted at 25 °C, with viscosity and refractive index values set based on water. Additionally, the DLS instrument was equipped with a 4 mW He/Ne laser light source (λ = 633 nm) at a 90° scattering angle. The intensity-averaged hydrodynamic diameters were calculated by a cumulant method and Mie theory. Transmission electron microscopy (TEM) images of BCNC samples were acquired using a JEM-1400 TEM instrument (JEOL). For sample preparation, 10 μL of a 0.1 mg/mL BCNC solution was applied to a carbon-coated copper grid and stained with ruthenium tetroxide. The average diameter of BCNC samples was measured from TEM images by using Image-Pro Plus software. The maximum absorption peak of Dox was determined at 485 nm using a UV–visible spectrophotometer (V-770/Jasco Inc.). Two calibration curves were generated using Dox in (i) phosphate-buffered saline (PBS) containing 0.5% (w/v) Tween 80 and (ii) dimethyl sulfoxide (DMSO), based on five concentrations and corresponding absorbance values. Fluorescence spectra of 1,1-dioctadecyl-3,3,3,3-tetramethylindotricarbocyanine iodide-loaded BCNC-3 (Dir-BCNC-3) were recorded by using a fluorescence spectrometer (F-4500/Hitachi) with an excitation wavelength of 754 nm.

### Materials

α-Methoxy-ω-hydroxyl polyethylene glycol (mPEG-OH, MW 2 kDa), 4-dimethylaminopyridine (DMAP; 99+%), lactide (LA; 98%), 1-[4-(2-hydroxyethoxy)-phenyl]-2-hydroxy-2-methyl-1-propanone (I2959; 98%), 2-(diethylamino)­ethanethiol hydrochloride (DEAET; >98%), 2,2-dimethoxy-2-phenylacetophenone (DMPA; 98%) were purchased from Sigma-Aldrich. Allyl-functional lactide (ALA) was synthesized following our previously reported method.^33^ Dichloromethane (DCM; HPLC), tetrahydrofuran (THF; HPLC), toluene (HPLC), diethyl ether (HPLC), acetone (HPLC), ethyl acetate (EA; HPLC), DMSO (HPLC), and DMF (HPLC) were purchased from J.T.Baker. EA, DCM, and DMF were dried through distillation over CaH_2_. LA was recrystallized from dried EA four times before usage. mPEG-OH was dried prior to use following this procedure: 1 mL of dried DCM was used to dissolve mPEG-OH, and the solvent was completely removed under vacuum. This process was repeated five times, followed by an additional five repetitions using toluene as the solvent. Dox·HCl (98%–102%) was obtained from Sigma-Aldrich and subsequently converted to hydrophobic Dox via HCl removal according to a previously reported protocol.^33^ Dir was purchased from MedChemExpress. All chemicals were used as received, unless otherwise stated.

### Synthesis of PEG-PLA-APLAs

The synthesis of PEG_45_-PLA_56_-APLA_42_ was employed as an example of the PEG-PLA-APLA preparation. A 10 mL reaction flask equipped with a magnetic stirring bar was preloaded with LA (864.0 mg; 6.0 mmol; 60 equiv), DMAP (48.8 mg; 0.4 mmol; 4 equiv), and mPEG-OH (MW 2 kDa, 200.0 mg; 0.1 mmol; 1 equiv). The flask was sealed and degassed under vacuum for 3 h, followed by filling with nitrogen gas. Subsequently, 5 mL of dry DCM was introduced to the flask to dissolve all the reactants for the first stage of ROP of LA, which was conducted at 35 °C for 72 h. At this point, approximately 90% of LA monomers had converted. Subsequently, ALA monomers (1020.0 mg; 6.0 mmol; 60 equiv) were added to the flask to conduct the ROP without stopping the ongoing reaction. After approximately 90% conversion of ALA monomers, the polymerization was terminated, and the resulting PEG_45_-PLA_56_-APLA_42_ was precipitated using cold ether. After vacuum drying, a white powder of PEG_45_-PLA_56_-APLA_42_ was obtained. ^1^H NMR (500 MHz, CDCl_3_, ppm): δ 1.42–1.59 (br m, C*H*_3_ units from LA and ALA), 2.58–2.77 (br m, C*H*_2_CH = CH_2_ units from ALA), 3.35 (s, terminal C*H*_3_O of mPEG-OH), 3.55–3.69 (br m, C*H*_2_O of mPEG-OH), 5.09–5.29 (br m, C*H*CH_3_ units from LA, C*H*CH_3_, C*H*CH_2_CH = CH_2_; and CH_2_CH = C*H*_2_ units from ALA), 5.69–5.84 (m, CH_2_C*H*=CH_2_ units from ALA). *M*_n_^NMR^ = 17.2 kDa, *M*_n_^GPC^ = 12.1 kDa, and PDI^GPC^ = 1.33. FTIR (cm^–1^): alkene C–H stretching 3082, C=O stretching 1750, C=C stretching 1643, C–H bending 1455, C–H stretching 1357, and C–O stretching 1085.

### Synthesis of PEG-PLA-CPLAs

Taking PEG-PLA-CPLA-80 as an example, PEG_45_-*b*-PLA_56_-*b*-APLA_42_ (100.00 mg; [ene]_0_ = 0.206 mmol, 1.0 equiv), the thiol-containing agent DEAET (34.79 mg; [SH]_0_ = 0.206 mmoL; 1.0 equiv), and DMPA (21.11 mg, 0.082 mmol, 0.4 equiv) were dissolved in 5 mL of chloroform in a flask to conduct a thiol–ene click reaction. The molar ratio of [ene]_0_:[SH of DEAET]_0_:[DMPA]_0_ was maintained at 1:1:0.4. The solution was degassed by three freeze–pumping–thaw cycles and subsequently irradiated with UV light (λ_max_ = 365 nm, 6 W, 0.12 A) for 30 min. The resulting product was purified by dialysis against acetone (MW cutoff: 3.5 kDa) for 5 days to remove unreacted DEAET and the photoinitiator. After complete vacuum drying, PEG-PLA-CPLA-80 was obtained. PEG-PLA-CPLA-50 was synthesized using the same procedure, except that the molar ratio of [ene]_0_:[SH of DEAET]_0_:[DMPA]_0_ was adjusted to 1:0.7:0.4. ^1^H NMR (500 MHz, CDCl_3_, ppm) of PEG-PLA-CPLA-80: δ 1.34–1.42 (br m, (C*H*_3_CH_2_)_2_NH^+^Cl^–^ from amine-functionalized units), 1.47–1.59 (br m, C*H*_3_ of units from LA and amine-functionalized units), 1.62–1.72 (br m, CH_2_C*H*_2_CH_2_SCH_2_ from amine-functionalized units), 1.90–2.13 (br m, C*H*_2_CH_2_CH_2_SCH_2_ from amine-functionalized units), 2.56–2.67 (br m, CH_2_CH_2_C*H*_2_SCH_2_ from amine-functionalized units), 2.92–3.26 (SC*H*_2_C*H*_2_NH^+^Cl^–^(C*H*_2_CH_3_)_2_ from amine-functionalized units), 3.34 (s, terminal C*H*_3_O of mPEG-OH), 3.57–3.65 (br m, C*H*_2_O of mPEG–OH), 5.05–5.24 (br m, C*H*CH_3_ of units from LA, C*H*CH_3_ and C*H*CH_2_CH_2_CH_2_S from amine-functionalized units). 5.69–5.84 (m, CH_2_C*H*=CH_2_ of units from amine-functionalized units). *M*_n_^NMR^ = 21.6 kDa, *M*_n_^GPC^ = 14.2 kDa, and PDI^GPC^ = 1.29. FTIR (cm^–1^): alkene C–H stretching 3083, N–H stretching 2250–2700, C=O stretching 1764, C=C stretching and OH resulting from environmental humidity 1649–1636, C–H bending 1458, C–H bending 1361, and C–O stretching 1089.

### Preparation of BCNCs and Therapeutics-Loaded BCNCs

BCNCs were prepared by an oil-in-water (O/W) nanoemulsion interfacial cross-linking method (Scheme 1c). Taking BCNC-3 as an example, PEG-PLA-CPLA-80 and CPLA-60 (6 mg; weight ratio 2:3) (Figure S1) were dissolved in a chloroform template (55, 80, or 110 μL), followed by dispersing the oil template into 4 mL of an aqueous solution containing 0.05 wt % I2959. The resulting milky O/W emulsion was ultrasonicated (Q125, Qsonica) at 50% amplitude for 30 min. After ultrasonication, the emulsion became transparent (Figure S2). The transparent emulsion was subsequently irradiated with UV light (λ_max_ = 365 nm, 6 W, 0.12 A) for 30 min to induce nanoemulsion interfacial cross-linking. After cross-linking, the chloroform template was removed by evaporation, and BCNC-3 was prepared. BCNC-1 and BCNC-2 were prepared similarly using PEG–PLA-CPLA-50 and PEG-PLA-CPLA-80, respectively, in the same amount as for BCNC-3.

**Scheme 1:**
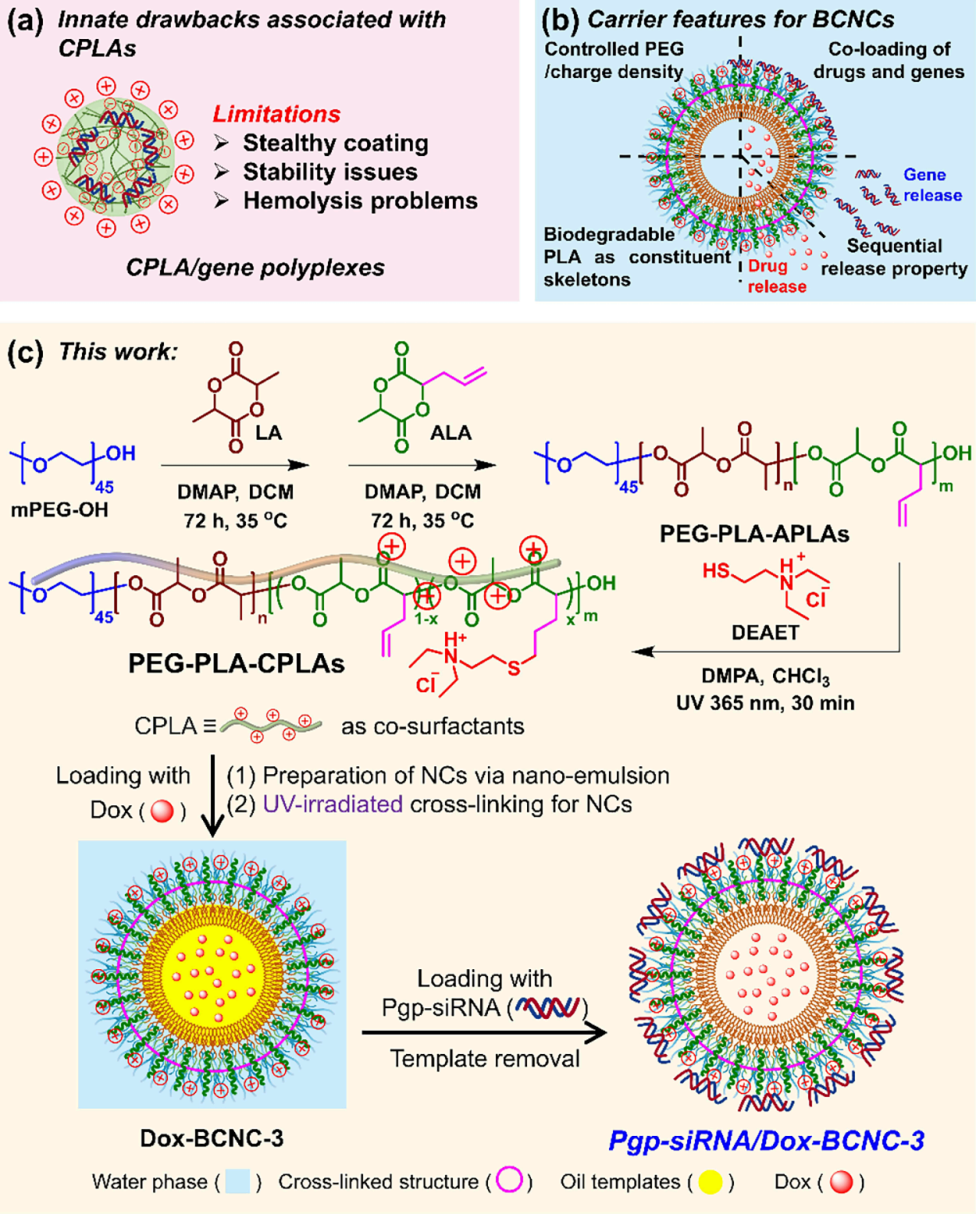
(a) Innate Drawbacks of CPLA Polymers when Used as Gene Vectors; (b) Design Concepts for BCNCs and Their Carrier Features; (c) Synthetic Route of PEG-PLA-CPLAs and the Loading of BCNCs with Solely Dox, Pgp-siRNA, or Pgp-siRNA/Dox Simultaneously^a^

a DMAP, 4-dimethylaminopyridine; CPLA, cationic polylactide; BCNC, biodegradable charged nanocapsules; PEG–PLA-CPLAs, poly­(ethylene glycol)-*b*-poly­(lactide)-*b*-poly­(cationic polylactide); Dox, doxorubicin; Pgp-siRNA, P-glycoprotein (Pgp) silencing gene.

For Dox-loaded BCNC-3 (Dox-BCNC-3), the same procedure was followed, except that 2 mg of Dox was preloaded into the chloroform template. The resulting Dox-BCNC-3 was dialyzed against 0.5% (w/v) Tween 80 in a PBS solution (MW cutoff: 3.5 kDa) for 4 h to remove unencapsulated Dox and residual I2959. After the dialysis, Dox-BCNC-3 was prepared. To determine the Dox loading content (LC) and encapsulation efficiency (EE), Dox-BCNC-3 was lyophilized and dissolved in DMSO, followed by measurement of the absorbance value at 485 nm on the UV–vis spectrophotometer. According to the Dox-DMSO calibration curve, the Dox loading amount was determined. Subsequently, the LC and EE of Dox-BCNC-3 were calculated using the following equations:

$$\mathrm{LC}\left(\mathrm{wt}\%\right)=\left(\frac{\mathrm{weight}\;\mathrm{of}\;\mathrm{loaded}\;\mathrm{Dox}}{\mathrm{weight}\;\mathrm{of}\;\mathrm{BCNC}-3}\right)\times 100\%$$

$$\mathrm{EE}(\mathrm{wt}\%)=\left(\frac{\mathrm{weight}\;\mathrm{of}\;\mathrm{loaded}\;\mathrm{Dox}}{\mathrm{weight}\;\mathrm{of}\;\mathrm{initial}\;\mathrm{Dox}\;\mathrm{feeding}}\right)\times 100\%$$

For the preparation of gene-loaded BCNCs, such as negative control siRNA (siNC)/Dox-BCNC-3, Pgp-siRNA/Dox-BCNC-3, siNC-BCNC-3, and Pgp-siRNA-BCNC-3, BCNC-3 with or without Dox was gently mixed in sterilized water, followed by enabling the solution to stand for 30 min as a definite polymer/siRNA weight ratio. Custom-synthesized siNC (sense: 5′-UUCUC CGAACGUGUCACGUTT-3′; antisense: 5′-ACGUGA CACGUUCGGAGAATT-3′), and Pgp-siRNA (sense: 5′-GAAACCAACUGUUAGUGUAdTdT-3′; antisense: 5′-UACACUGACAGUUGGUUUCdTdT-3′) were purchased from Guangzhou Ribobio Co., Ltd. These siRNA molecules can be labeled with fluorescein amidites (siFAM) at the 5′ end for fluorescence detection using a fluorescent microscope or flow cytometry.

### Colloidal Stability of BCNCs

To evaluate the colloidal stability of BCNC samples, the samples were dispersed in pH 7.4 PBS at a concentration of 1 mg/mL and incubated at 37 °C with mild shaking. At specific time points (1, 3, 5, and 7 days), particle size changes were monitored using DLS. Additionally, to mimic physiological conditions, the BCNC samples (1 mg/mL) were further diluted 1000-fold in pH 7.4 PBS to a final concentration of 1 × 10^–3^ mg/mL and dispersed in a 0.1 M NaCl solution and bovine serum albumin (BSA; Sigma-Aldrich; 1 mg/mL) solutions. These suspensions were incubated at 37 °C, followed by the DLS measurements at 1 to 7 days for acquiring the particle size changes of BCNC samples. Furthermore, the storage stability of BCNC-relevant samples was evaluated in pH 7.4 PBS at 25 °C for 7 days.

### Degradation Behavior of BCNCs

The degradability of the major constituent polymers, PEG-PLA-CPLA-80 and CPLA-60 for BCNCs, was measured by GPC. In the measurement, 3 mg of each polymer was dissolved in 1 mL of PBS at pH 7.4 and pH 5.5 and stirred gently at 37 °C. At predetermined time points, 1 mL aliquots were collected, and the solvent was completely removed under vacuum. The residual polymers were subsequently dissolved in DMF for the GPC analysis.

### Agarose Gel Retardation Assay for siRNA-Loaded BCNCs

To determine the minimum amount of BCNC-3 required for complete siRNA encapsulation, we performed agarose gel electrophoresis was performed. The siRNA amount was fixed at 10 pmol (0.133 μg), followed by mixing it with BCNC-3 at polymer/siRNA weight ratios of 1:1, 2:1, 4:1, 8:1, 16:1, 32:1, and 64:1. The mixtures were allowed to stand at room temperature for 30 min, followed by the addition of an appropriate amount of 5× loading buffer. Samples were loaded onto a 1 wt % agarose gel containing GelRed and electrophoresed at 100 V for 15 min. The gel was visualized under a UV transilluminator (FluorChem E/ProteinSimple), and the gray values of the bands were analyzed by using ImageJ software.

### Serum Enzymatic Protection Assay for siRNA-Loaded BCNCs

To evaluate the stability of Pgp-siRNA-BCNC-3 against enzymatic degradation, RNase A (No. 10109169001, Sigma-Aldrich) was used to mimic serum RNA-degrading enzymes in vivo. Pgp-siRNA-BCNC-3 was incubated in RNase A solution (0.25 μg/μL) at 37 °C for 30 min to 12 h. After incubation, 1 wt % sodium dodecyl sulfate (SDS) was added for 5 min at 4 °C to detach the adsorbed Pgp-siRNA from the BCNCs. The released siRNA was subsequently electrophoresed at 100 V for 15 min, visualized under a UV transilluminator, and analyzed using ImageJ software.

### In Vitro siRNA and Dox Release from Therapeutics-Loaded BCNCs

To obtain the Pgp-siRNA release profiles of Pgp-siRNA-BCNC-3 with or without the Dox loading, a siRNA release assay was performed.^49^ In this assay, the Pgp-siRNA-loaded NCs were dispersed in a 96-well plate, and each well was filled with 10× its volume of PBS buffer (pH 5.5 or 7.4). Sequentially, the plate was shaken at 100 rpm at 37 °C. At predetermined release times, the solution in each well was removed and centrifuged at 4 °C at 13,000 rpm for 5 min, followed by measuring the siRNA concentration in the supernatant (2.5 μL) using a micro nucleic acid quantification instrument (EPOCH 2/BioTek). The siRNA concentrations of naked siRNA and a no added sample (processed using the same procedure) were used as the standard and the blank control, respectively. As a result, the percentage of siRNA release from Pgp-siRNA-BCNC-3, with or without Dox loading, at each release time point was calculated by dividing the siRNA concentration in the supernatant by that of the corresponding naked siRNA.

To determine the Dox release profiles from Dox-loaded BCNC samples, a dialysis bag (MW cutoff: 3.5 kDa) containing those samples was prepared and placed in 45 mL of PBS solution (pH 7.4) containing 0.5% (w/v) Tween 80 as the release medium. The medium was maintained at 37 °C with gentle stirring. At specific time intervals, 3 mL of the release medium was withdrawn for UV–vis measurements and replaced with an equal volume (3 mL) of fresh PBS solution. Based on the calibration curve of Dox in PBS solution containing 0.5% (w/v) Tween 80, the Dox concentrations at specific time points were calculated. Accordingly, the Dox release profiles were obtained.

### Cell Culture and Cytotoxicity Assessment

To evaluate the biocompatibility of BCNCs and the cytotoxicity of therapeutics-loaded BCNCs, MCF-7 (breast cancer cell line), MCF-7/ADR (multidrug-resistant human breast cancer cell line), HepG2 (hepatocellular carcinoma cell line), HeLa (human cervical cancer cell line), and MES-SA/Dx5 (multidrug-resistant human uterine sarcoma cell line) were used for study. A 3-(4,5-dimethylthiazol-2-yl)-diphenyl tetrazolium bromide (MTT) colorimetric assay was employed for the assessment. Cells were cultured in Dulbecco’s modified Eagle medium/nutrient mixture F-12 (DMEM/F-12; Gibco) supplemented with 10% fetal bovine serum (Gibco) and 1% penicillin–streptomycin (Gibco) at 37 °C in a 5% CO_2_-humidified atmosphere.

Under these culture conditions, cells were seeded into 96-well plates at a density of 5 × 10^3^ cells/well and incubated for 24 h. Subsequently, BCNCs were added at concentrations of 10, 20, 40, 80, 160, 320, and 640 μg/mL, followed by incubation for 48 h. Untreated cells served as the control group. After incubation, 20 μL of an MTT solution (5 mg/mL) was added to each well, and the plates were incubated for 4 h. Subsequently, the medium was removed, and 150 μL of DMSO was added to dissolve the MTT formazan crystals. The plate was shaken for 15 min to ensure complete mixing. Ultimately, the optical density (OD) of each well was measured at 570 nm using a Microplate Reader (800TS/BioTek), with 630 nm as the reference wavelength. Cell viability was calculated using the following equation.

$$\mathrm{cellviability}(\%)=\frac{{\mathrm{OD}}_{\mathrm{sample}}-{\mathrm{OD}}_{\mathrm{blank}}}{{\mathrm{OD}}_{\mathrm{control}}-{\mathrm{OD}}_{\mathrm{blank}}}\times 100\%$$

In the formula, OD_blank_, OD_sample,_ and OD_control_ represent the OD values of the solvent for the used culture medium, the sample-treated culture medium, and the untreated culture medium, respectively.

Using the same cell culture procedures, therapeutics-loaded BCNCs were tested for their antitumor efficacy based on the corresponding cell viability. The loaded therapeutics included Dox, siNC, and Pgp-siRNA.

### Intracellular Uptake of Therapeutics-Loaded BCNCs

The cellular uptake and intracellular release of therapeutics-loaded BCNCs were evaluated through confocal laser scanning microscopy (CLSM; TSC-SP5II/Leica) and flow cytometry (FACSAria/Becton Dickinson). For CLSM observation, the concentrations of loaded or free therapeutics, such as Dox and siFAM, were set at 5 μg/mL and 50 nmol/L, respectively. MCF-7 and MCF-7/ADR cells were seeded at a density of 1 × 10^5^ cells/well and incubated for 24 h. Subsequently, the culture medium was replaced with a serum-free medium containing therapeutics-loaded BCNCs. After a specified incubation period, cells were fixed with 4% paraformaldehyde for 30 min and stained with 4′,6-diamidino-2-phenylindole (DAPI) at 50 μg/mL to visualize the nucleus. During CLSM observation, filter settings were as follows: FAM was excited with a 488 nm laser, and emission was collected with a 525/50 nm band-pass filter. Dox was excited by a 540 nm laser, and the emission was gathered with a 605/50 nm band-pass filter. To monitor endolysosomal escape of therapeutics-loaded BCNCs, cells were stained with LysoTracker Red to label endosomal and lysosomal compartments, with the nucleus exhibiting red fluorescence after staining.

Flow cytometry was used to quantify the fluorescence intensity of therapeutics-loaded BCNC-treated cells. For this assay, MCF-7 and MCF-7/ADR cells were seeded in six-well plates at a density of 1 × 10^6^ cells/well and incubated for 24 h. Subsequently, the medium was replaced with a serum-free medium containing therapeutics-loaded BCNCs. After the incubation period, cells were harvested using trypsin and centrifuged at 1200 rpm for 10 min. The collected cells were washed three times with PBS (pH 7.4) before flow cytometry analysis. Fluorescence intensity was measured by using the signals from Dox and FAM labels.

### Gene Transcription Level Assay

The intracellular Pgp mRNA level was measured in accordance with our previous report.^43,49^ MCF-7 and MCF-7/ADR cells (1 × 10^5^ cells/well) were seeded in six-well plates and cultured at 37 °C in a humidified atmosphere containing 5% CO_2_ for 24 h. After cultivation, cells were treated with Pgp-siRNA, siNC-loaded BCNCs, or naked siRNA molecules. Following 48 h of incubation, cells were harvested using trypsin and washed twice with pH 7.4 PBS. Total RNA was extracted from the treated cells using TRIzol reagent (No. 15596026/Invitrogen) according to the manufacturer’s protocol. Subsequently, 1 μg of total RNA was reverse-transcribed into cDNA using the QuantiTect Reverse Transcription Kit (No. 205311, Qiagen) according to the instructions from the supplier. The mRNA level of the Pgp gene was further quantified by quantitative real-time polymerase chain reaction (qRT-PCR) using a QuantStudio 1 system (Applied Biosystems) with Bestar Sybr Green qPCR Master Mix (No. DBI-2043, DBI Bioscience). Gene expression was normalized to β-actin, which served as an internal control. The primer sequences were as follows: Pgp-F: 5′-CCATAGCTCGTGCCCTTGTTAGA-3′, Pgp-R: 5′-CGGTGAGCAATCACAATGCAG-3′. β-actin-F: 5′-CTCCATCCTGGCCTCGCTGT-3′, β-actin-R: 5′-GCTGTCACCTTCACCGTTCC-3′.

### Western Blotting Analysis

The Pgp silencing efficacy of Pgp-siRNA-loaded BCNCs was assessed by Western blot. MCF-7/ADR and MES-SA/Dx5 cells were seeded in six-well plates at a density of 2 × 10^5^ cells per well. Cells were treated with 2 mL of fresh culture medium containing the corresponding formulations (final Pgp-siRNA concentrations: 50 nM per well) and incubated for 48 h. After incubation, cells were harvested by trypsinization, centrifuged, and washed three times with cold PBS (pH 7.4). Subsequently, the cells were resuspended, homogenized, and lysed in RIPA buffer (No. 89901, Thermo Fisher Scientific), containing 1 mM phenylmethanesulfonylfluoride. Protein extracts were obtained by centrifugation, and protein concentrations were determined using a bicinchoninic acid protein assay (Pierce Biotechnology). Equal amounts of protein were separated by SDS-polyacrylamide gel electrophoresis and transferred onto polyvinylidene difluoride membranes, which were blocked with TBST buffer containing 5% (v/v) BSA for 60 min at room temperature. Membranes were incubated overnight at 4 °C with primary monoclonal antibody against Pgp (C219, GeneTex; 1:500 dilution in TBST buffer with 5% BSA). For internal controls, anti-β-actin antibody (A2228, Sigma-Aldrich) was used for MCF-7/ADR cells, and the antiglyceraldehyde 3-phosphate dehydrogenase (GAPDH) antibody (G9545, Sigma-Aldrich) was used for MES-SA/Dx5 cells. After washing, membranes were incubated with goat antimouse IgG-peroxidase-linked secondary antibody (1:2000 dilution in TBST buffer with 5% BSA) at room temperature for 60 min. Protein signals were visualized using an ECL kit (Nos. 1863096 and 1863097, Thermo Fisher Scientific) and the Pico Plus Detection Kit (Thermo Fisher Scientific). Images were acquired using the ChemiDoc MP Imaging System (Bio-Rad) and analyzed with ImageLab Software (Bio-Rad).

### Animals and Tumor Models

BALB/c nude mice (20 ± 2 g, 6 weeks old) were purchased from the National Laboratory Animal Center (Taipei, Taiwan). To establish an orthotopic MCF-7/ADR tumor model, MCF-7/ADR cells (3 × 10^6^) in 100 μL of a mixture consisting of PBS and matrigel 1:1 (w/w) were injected into the right mammary fat pads of the mice. At the end of the experiments, all of the mice were euthanized via CO_2_ inhalation. All animal experiments related to biodistribution and toxicity studies were conducted in accordance with the Guidelines for the Care and Use of Laboratory Animals of Kaohsiung Medical University and were approved by the Institutional Animal Care and Use Committee (IACUC) of Kaohsiung Medical University (IACUC Approval No. 110141). The antitumor efficacy experiments were performed in accordance with the Regulations for the Administration of Affairs Concerning Experimental Animals of the People’s Republic of China and were approved by the Animal Ethical and Welfare Committee of Shenzhen University (AEWC-SZU) (Ethics Approval No. AEWC-A202401956).

### Statistical Analysis

Quantitative data are presented as the mean ± standard deviation (SD) and were analyzed using one-way or two-way ANOVA followed by Tukey’s post hoc tests, using GraphPad Prism software. A *p*-value <0.05 was considered statistically significant (*p < 0.05, **p < 0.01, ***p < 0.001).

## Results and Discussion

### Synthesis and Characterization of PEG-PLA-CPLAs

CPLA, a tertiary amine-functionalized PLA, has been previously synthesized by our group (Figure S1) and successfully used as a promising gene delivery carrier for targeting various cancer cells due to its high gene delivery efficiency, biodegradability, biosafety, and rapid degradation.^27,38–42^ Nonetheless, the absence of a stealth coating on the resulting CPLA/gene polyplexes resulted in low stability under physiological-mimicking conditions and raised hemolysis concerns (Scheme 1a).^50^ Moreover, particulate properties, such as size and structure of the polyplexes, are difficult to control.^48,51^ In this context, a new triblock copolymer, PEG-PLA-CPLAs, was prepared to generate well-defined BCNCs (Scheme 1b). The additional PEG and PLA blocks are expected to improve colloidal stability and enhance the coloading capacity for both hydrophobic and hydrophilic therapeutics. Additionally, gene payloads located in the CPLA interfacial layer of the NCs are anticipated to be released faster than hydrophobic anticancer drugs from the PLA core to the faster degradation rate of CPLA relative to PLA.^48^ Based on this NC structure design, PEG-PLA-CPLAs were synthesized by a sequential ROP procedure and a subsequent thiol–ene postmodification. Subsequently, the copolymers were converted into NCs using CPLAs as a cosurfactant through an O/W nanoemulsion cross-linking process (Scheme 1c). The resulting NCs are referred to as BCNCs. Following single-loading or coloading with hydrophobic Dox and Pgp-siRNA, the NCs are termed Dox-BCNCs, Pgp-siRNA-BCNCs, or Pgp-siRNA/Dox-BCNCs.

To synthesize PEG-PLA-CPLAs, the precursor PEG-PLA-APLAs were prepared using mPEG-OH (MW = 2 kDa; number-average degree of polymerization (DP_*n*_) = 45) and DMAP as the initiator and the organocatalyst in a sequential ROP procedure, with LA and ALA as the monomers at the first and second stages, respectively (Scheme 1c and Table S1). In the first stage of the ROP of LA monomers ([mPEG-OH]_0h_:[DMAP]_0h_:[LA]_0h_ = 1:4:60; 35 °C, 72 h, in DCM; approximately 90% conversion of LA), PLA was successfully covalently attached to the ω-chain end of the mPEG-OH to form PEG-*b*-PLA-OH. Subsequently, the second-stage ROP of ALA monomers was performed without interrupting the ongoing reaction ([mPEG-OH]_72h_:[DMAP]_72h_:[ALA]_72h_ = 1:4:60; 35 °C, 72 h, in DCM; approximately 90% conversion of ALA monomers). The resulting polymer, termed PEG_45_-*b*-PLA_56_-*b*-APLA_42_ (the subscript number denoting the DP_*n*_ value of the corresponding repeating unit), was successfully synthesized, and its well-defined chemical structure was thoroughly characterized by ^1^H NMR, FTIR, and GPC (Figure 1). According to the ^1^H NMR result (Figure 1a), the DP_*n*_ of the PLA blocks (56) in PEG_45_-*b*-PLA_56_-*b*-APLA_42_ was calculated by comparing the resonance intensities of the C*H* protons from ALA and LA monomers and the allyl C*H*_2_ protons (CH = C*H*_2_) from ALA at 5.09–5.29 ppm with the resonance intensities of the 180 protons from C*H*_2_C*H*_2_O units of mPEG-OH at 3.55–3.69 ppm. The DP_*n*_ of the APLA block (42) was determined by comparing the allyl CH protons (C*H*=CH_2_) from units of ALA at 5.69–5.84 ppm with the same reference protons from mPEG-OH. The IR spectra also revealed multiple absorption peaks consistent with the chemical structure of PEG_45_-*b*-PLA_56_-*b*-APLA_42_ (Figure 1b), including 3082 cm^–1^ (C=C from the ALA units), 1750 cm^–1^ (ester bonds from both the ALA and LA units), 1643 cm^–1^ (C=C from the ALA units), 1455 cm^–1^ (methylene groups from the ALA units), 1357 cm^–1^ (methyl groups from both the ALA and LA units), and 1085 cm^–1^ (ether bonds from the PEG block). GPC results showed that PEG_45_-*b*-PLA_56_-*b*-APLA_42_ exhibited a *M*_n_^GPC^ value of 12.1 kDa and a PDI value of 1.33 (Figure 1c). Compared with mPEG-OH eluted at 32.1 mL, PEG_45_-*b*-PLA_56_-*b*-APLA_42_ exhibited a smaller elution volume of 27.1 mL, indicating that the PLA and the APLA blocks were successfully and sequentially grown on the ω-chain end of mPEG-OH. Additionally, the monomodal molecular weight distribution and low PDI further demonstrated the living character of the ROP procedure. However, a small shoulder peak at 29.1 mL was observed. The peak was attributed to residual PLA homopolymers initiated by trace amounts of water or impurities, which could not be completely fractionated.^52^

**Figure 1:**
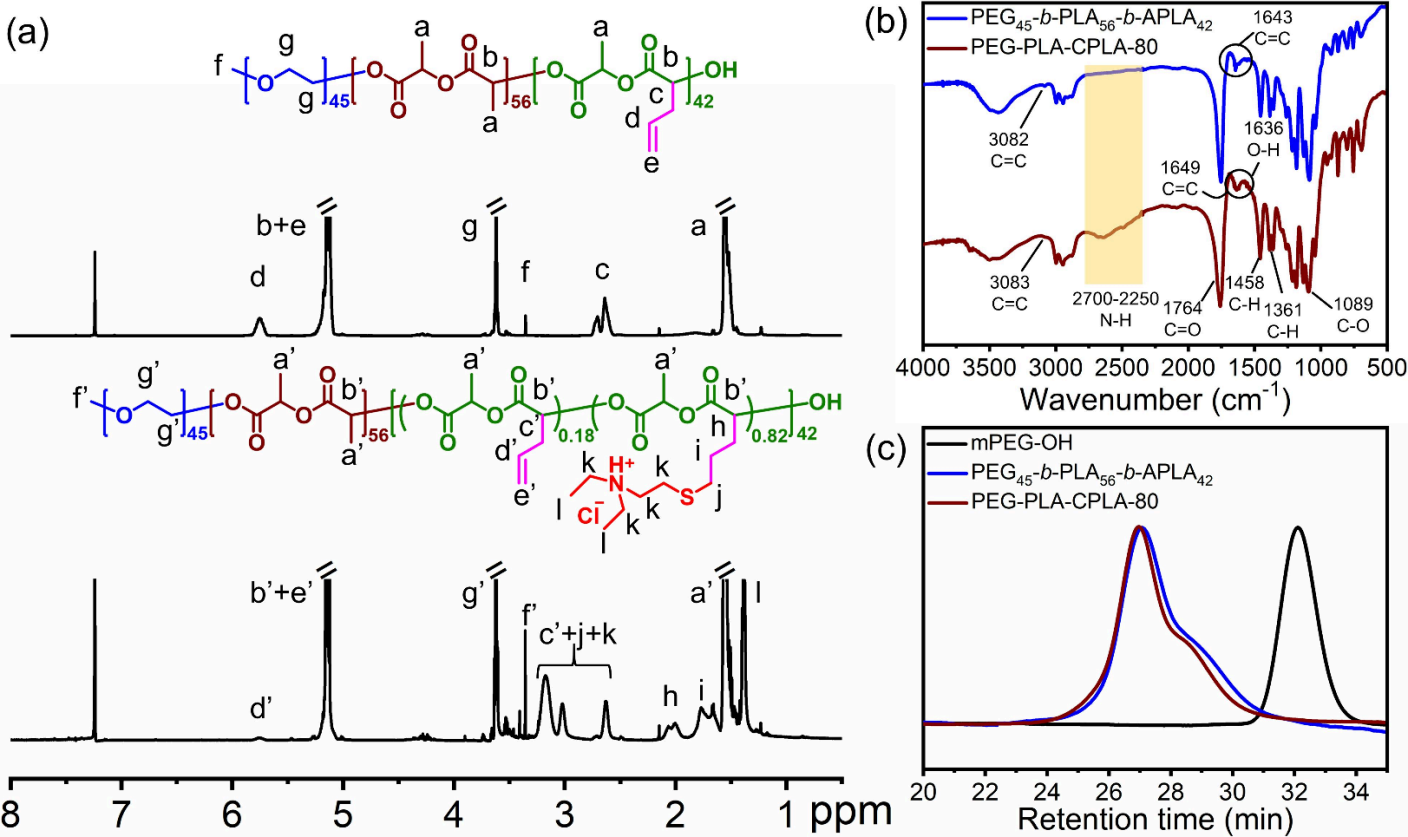
(a) 500 MHz ^1^H NMR spectra of PEG_45_-*b*-PLA_56_-*b*-APLA_42_ (upper panel) and PEG-PLA-CPLA-80 (lower panel) in CDCl_3_; (b) FTIR spectra of PEG_45_-*b*-PLA_56_-*b*-APLA_42_ and PEG–PLA-CPLA-80; and (c) GPC curves of mPEG–OH (MW = 2.0 kDa), PEG_45_-*b*-PLA_56_-*b*-APLA_42,_ and PEG-PLA-CPLA-80.

Serving as the precursor polymer for PEG-PLA-CPLAs, PEG_45_-*b*-PLA_56_-*b*-APLA_42_ has undergone postmodification treatment of its APLA block. The APLA block was converted into cationic LA (CLA) units by converting the allyl groups into tertiary amine moieties through a controlled UV-induced thiol–ene functionalization (Scheme 1c). Using DEAET as the thiol-containing agent and DMPA as the photoinitiator, the thiol–ene click reaction was performed under UV irradiation (λ = 365 nm) at room temperature for 30 min. After unreacted agents were removed, PEG_45_-*b*-PLA_56_-*b*-poly­(ALA_0.53_-*co*-CLA_0.47_)_42_ (PEG-PLA-CPLA-50) and PEG_45_-*b*-PLA_56_-*b*-poly­(ALA_0.2_-*co*-CLA_0.8_)_42_ (PEG-PLA-CPLA-80), exhibiting charge densities of 47.0 and 82.1%, respectively, were obtained by controlling the molar ratio of [ene]_0_:[SH]_0_:[DMPA]_0_ during functionalization (Table 1). Taking PEG-PLA-CPLA-80 as an example (Figure 1a), the mole fraction of CLA units relative to the original APLA block was calculated to be 82% by comparing the resonance intensities of allyl C*H* protons (C*H*=CH_2_) of ALA units at 5.69–5.84 ppm with the methyl CH_2_C*H*_3_ protons of CLA units at 1.34–1.42 ppm. This result indicates that the charge density of PEG-PLA-CPLAs can be precisely controlled by adjusting the reaction conditions during thiol–ene modification. Accordingly, an optimal charge density can be imparted to PEG-PLA-CPLAs to balance transfection efficiency and the toxicity. Moreover, the allyl functionality of the original APLA block can be preserved and utilized for further chemical modification such as cross-linking of PEG-PLA-CPLAs. In addition to the ^1^H NMR result, IR analysis further confirmed the well-defined chemical structure of PEG-PLA-CPLAs (Figure 1b). Using PEG-PLA-CPLA-80 as an example, the IR absorption spectrum displayed several characteristic absorption peaks relating corresponding to its chemical structure, including, 2250–2700 cm^–1^ (amine salts from CLA units), 1764 cm^–1^ (ester bonds from both ALA and LA units), 1649–1636 cm^–1^ (C=C from ALA units and OH from the environment humidity), 1458 cm^–1^ (methylene groups from ALA units), 1361 cm^–1^ (methyl groups from both ALA and LA units), and 1089 cm^–1^ (ether bonds from the PEG block). GPC results showed that PEG-PLA-CPLA-80 had a weight distribution curve similar to that of the precursor polymer PEG_45_-*b*-PLA_56_-*b*-APLA_42_, with a slightly smaller elution volume (26.9 mL) (Figure 1c). Due to the original peak of PEG_45_-*b*-PLA_56_-*b*-APLA_42_, PEG-PLA-CPLA-80 retained a minor residual peak. The *M*_n_^GPC^ and PDI were determined to be 14.2 and 1.29,kDa, respectively, indicating that the incorporation of tertiary amine moieties via thiol–ene modification on the precursor polymer backbone had no significant effect on the hydrodynamic volume. Importantly, undesired reactions, such as cross-linking, were completely avoided during the thiol–ene functionalization.^48^

**Table 1:** Synthesis and Characterization of PEG-PLA-CPLAs through the Thiol–Ene Modification of PEG_45_-*b*-PLA_56_-*b*-APLA_42_

entry^a^	[ene]:[SH]:[DMPA]^b^	amine mol %^c^	*M*_n_^NMR^ (kDa)^c^	*M*_n_^GPC^ (kDa)^d^	PDI^d^
PEG-PLA-CPLA-50	1:0.7:0.4	47.0	19.8	12.3	1.21
PEG-PLA-CPLA-80	1:1: 0.4	82.1	21.6	14.2	1.29

a The numbers after the final dash lines indicate the approximate amine substitution percentage of the CPLA block of the corresponding polymer entry.

b Thiol–ene modification condition: UV irradiation (λ_max_ = 365 nm) for 30 min using chloroform as the reaction solvent;

c Calculated from^1^H NMR.

d Relative to linear polystyrene standards, using DMF as the eluent. DMF, *N*,*N*-dimethylformamide; NMR, nuclear magnetic resonance; CPLA, cationic polylactide.

### Preparation of Well-Defined BCNCs via PEG-PLA-CPLAs

With hydrophilic PEG and CPLA segments on the two side blocks and a hydrophobic PLA segment in the middle, PEG-PLA-CPLAs were designed as a new degradable amphiphilic polymeric surfactant capable of directly forming O/W nanoemulsions without the need for nondegradable or toxic small-molecule surfactants (Scheme 1c). Following the formation of transparent O/W nanoemulsions, the PEG-PLA-CPLAs are expected to orient their two hydrophilic segments toward the aqueous phase, while the hydrophobic PLA segment points inward toward the oil template. Accordingly, our nanoemulsion interfacial cross-linking technique can be used to covalently bond the PEG-PLA-CPLAs at the O/W interfaces via radical chain polymerization. Due to the use of oil templates and the balanced interactions among the three segments at the O/W interfaces, PEG-PLA-CPLAs can form cross-linked BCNCs after interfacial cross-linking and removal of the oil template.

During the nanoemulsion interfacial cross-linking, PEG-PLA-CPLAs, chloroform, and I2959 were used as the polymeric surfactant, the oil template, and the photoinitiator, respectively (Table 2). Two PEG-PLA-CPLAs with charge densities of 47.0 and 82.1% (PEG-PLA-CPLA-50 and PEG-PLA-CPLA-80) were used to prepare BCNC-1 and BCNC-2, respectively. With a surfactant/oil template ratio of 0.04 and an oil/water weight ratio of 24, a transparent O/W nanoemulsion was obtained after 30 min ultrasonication by combining the three components (Figure S2). Taking BCNC-2 as an example, IR analysis showed that the adsorption peaks associated with the original allyl functionality of the precursor PEG-PLA-CPLA-80 at 993, 1643, and 3082 cm^–1^ have disappeared after UV treatment (λ = 365 nm) for 30 min under the transparent emulsion conditions (Figure 2a).^53^ This indicates that PEG-PLA-CPLAs can be cross-linked through UV-irradiated nanoemulsion cross-linking. In addition to forming the cross-linked structure, it was observed that the particle size of BCNCs can be effectively tuned by controlling the amount of the oil template and the charge density of the BCNCs (Table 2). For BCNC-1, increasing the oil template volume from 55 to 110 μL resulted in an increase in particle size from 144 to 261 nm. In contrast, BCNC-2 showed less pronounced size changes with variation in the oil template volume; at 110 μL, BCNC-2 had a particle size of 80 nm, compared with 261 nm for BCNC-1 (Figure 2b). The zeta potential values of BCNC-1 and BCNC-2 were 21.8 and 25.7 mV, respectively (Table 2). Considering its smaller particle size and higher zeta potential, BCNC-2 was selected as the base formulation for coloading drugs and genes due to its favorable surfactant effect and particulate properties. Given the chemical structure of the precursors, BCNCs are expected to have a high PEG segment density on the capsular surface, which may impede the adsorption of gene payloads.^54^ To address this, BCNC-3 was prepared using a mixture of PEG-PLA-CPLA-80 and CPLA-60 (a CPLA polymer with a charge density of 60%, Figure S1) at a 2:3 weight ratio. Compared with BCNC-2, BCNC-3 exhibited an enhanced zeta potential value of 38.1 mV and a larger particle size of 162 nm at the same oil template volume of 110 μL (Table 2 and Figure 2b). These results demonstrate that the particle size of BCNCs can be readily tuned by adjusting the oil template volume during nanoemulsion interfacial cross-linking. Additionally, both the PEG segment density and the zeta potential of BCNCs can be modulated by incorporating CPLAs as the cosurfactant during the preparation procedure.

**Figure 2:**
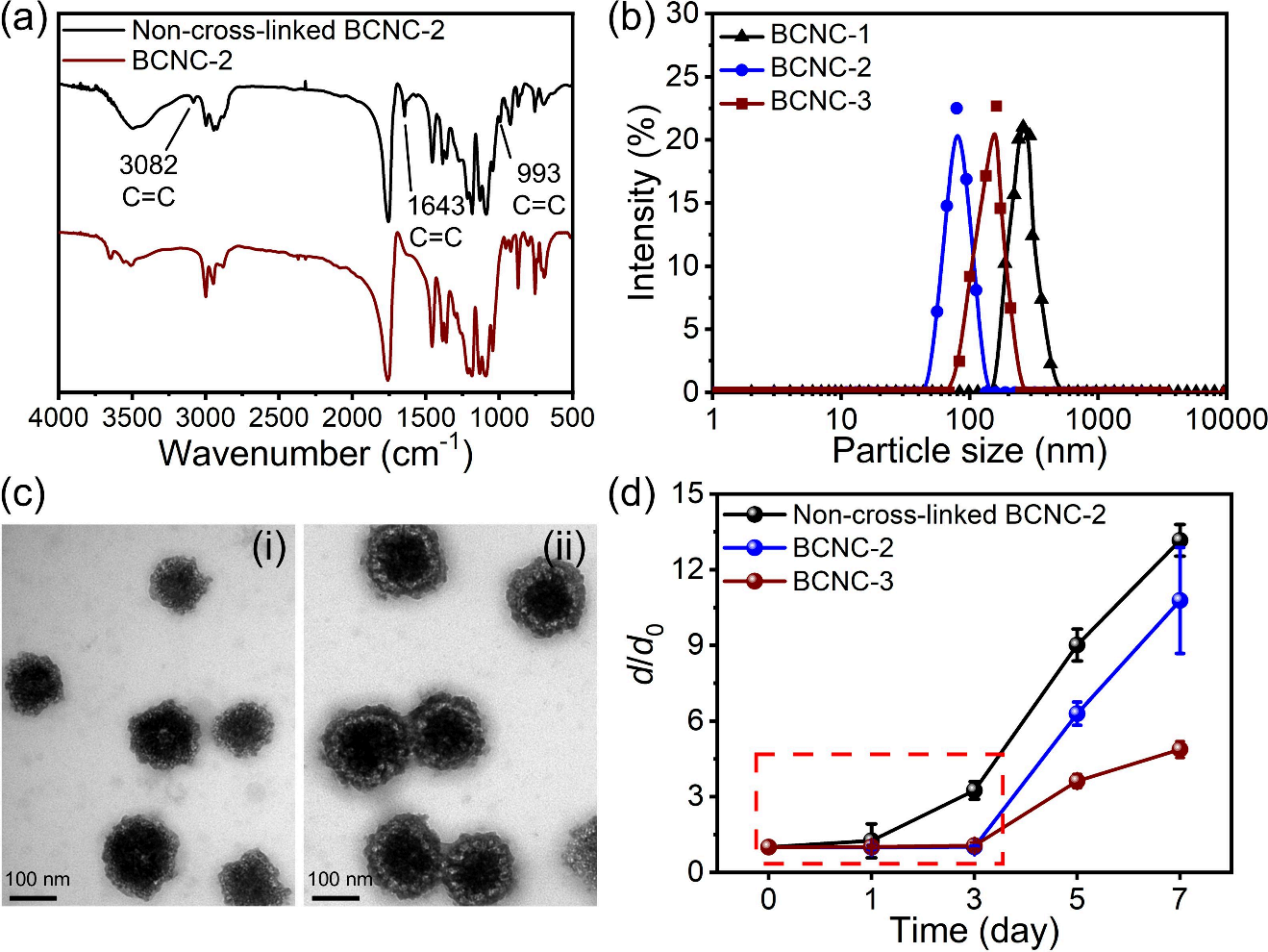
(a) FTIR spectra of non-cross-linked BCNC-2 and BCNC-2. (b) DLS results of BCNCs. (c) TEM images of (i) BCNC-2 and (ii) BCNC-3. (d) *d*/*d*_0_ values of BCNCs resulting from 110 μL of the oil template at predetermined incubation times in PBS (pH 7.4) at 37 °C. Error bars represent standard deviations from three independent experiments.

**Table 2:** Preparation of BCNCs via an Interfacial Nanoemulsion Cross-linking Procedure Using PEG-PLA-CPLAs and CPLAs as the Polymeric Surfactant and Cosurfactant

	BCNC-1^b^ ^,^ ^c^		BCNC-2^b^ ^,^ ^c^		BCNC-3^b^ ^,^ ^c^	
oil template volume (μL)^a^	*D*_h,i_ (nm)/PDI	zeta potential (mV)	*D*_h,i_ (nm)/PDI	zeta potential (mV)	*D*_h,i_ (nm)/PDI	zeta potential (mV)
55	144 ± 7.8/0.272 ± 0.03	23.0 ± 1.3	66 ± 4.2/0.245 ± 0.01	28.2 ± 1.6	110 ± 7.3/0.240 ± 0.02	37.4 ± 1.3
80	180 ± 10.2/0.240 ± 0.02	23.6 ± 1.0	71 ± 6.8/0.274 ± 0.05	27.3 ± 1.0	127 ± 9.2/0.395 ± 0.01	38.3 ± 0.2
110	261 ± 7.5/0.276 ± 0.05	21.8 ± 1.0	80 ± 4.2/0.291 ± 0.04	25.7 ± 1.9	162 ± 5.4/0.393 ± 0.04	38.1 ± 0.1

a All BCNCs were prepared at a surfactant/oil template weight ratio of 0.04 and an oil/water weight ratio of 24. For BCNC-1 and BCNC-2, only PEG-PLA-CPLAs were used in the emulsification process. For BCNC-3, the PEG-PLA-CPLA-80/CPLA-60 weight ratio was adjusted to 2:3.

b The polymeric surfactants used for preparing BCNC-1 and BCNC-2 were PEG-PLA-CPLA-50 and PEG-PLA-CPLA-80.

c The hydrodynamic diameter (*D*_h,i_) and zeta potential of the BCNCs were measured five times by DLS to obtain the average values, and the error bars were calculated from five independent experiments.

Compared with PEI, a widely used polymeric gene transfection agent, BCNCs can adjust the surface potential from 21.8 to 38.3 mV.^55^ This is because the constituent polymers, including PEG-PLA-CPLAs and CPLAs, are capable of tuning the amount of tertiary amine bearing on the side chains. The tertiary amine is the positive charge source for PEG-PLA-CPLAs and CPLAs. As a result, the toxicity caused by excessive positive charges from polymers such as PEI, a highly charged polymer, can be avoided in BCNCs.^56^ In addition, current PEGylated drug/gene codelivery carriers developed to overcome MDR have limited ability to regulate surface PEG density because their buildings blocks are solely PEG-based copolymers.^57^ In contrast, BCNC-3, composed of PEG-PLA-CPLA-80 and CPLA-60 as well as PEGylated and non-PEGylated cationic PLAs, can adjust the PEGylated density on the particle’s surface. It was reported that the PEG density on the carrier surface significantly affected the gene adsorption capacity, binding ability with cell membranes, and cell uptake efficiency.^58^ Correspondingly, the ability to adjust the PEG density by using PEGylated and non-PEGylated cationic PLAs as the constituent polymers enables BCNC-3 to alleviate the innate limitations associated with PEGylation.

TEM analysis confirmed that both BCNC-2 and BCNC-3 exhibit well-defined capsular structures, likely consisting of a PLA core surrounded by a PEG/CPLA shell, based on contrast differences (Figure 2c). Importantly, incorporation of CPLA to adjust the PEG segment density of BCNCs did not affect the capsular morphology. Based on the TEM images, BCNC-2 and BCNC-3 exhibited *D*_av_ values of 131 and 170 nm, respectively, which were consistent with their corresponding DLS measurements. To investigate the therapeutic-loading effect on the BCNC particulate properties, BCNC-3 was preloaded with Dox and Pgp-siRNA/Dox. The resulting Dox-BCNC-3 and Pgp-siRNA/Dox-BCNC-3 morphologies were observed by TEM (Figure S3). The TEM images revealed that BCNC-3 retained its original capsular morphology after the loading with Dox and Pgp-siRNA/Dox. The calculated *D*_av_ values were 134 and 138 nm for Dox-BCNC-3 and Pgp-siRNA/Dox-BCNC-3, respectively, indicating that the loading of therapeutic agents has no significant effects on the capsular morphology or particle size of BCNC-3. In addition, DLS results showed that the particle sizes and zeta potential of Dox-BCNC-3 and Pgp-siRNA/Dox-BCNC-3 were comparable to those of BCNC-3 (Table S2). Based on the TEM and DLS results, it was considered that the loading of therapeutics had no appreciable influence on the particulate properties.

To demonstrate the colloidal stability of BCNC-2 and BCNC-3, the samples were incubated in PBS (pH 7.4) at 37 °C for 7 days, and their particle sizes were monitored daily by DLS (Figure S4). The particle size change was expressed as the ratio *d*/*d*_0_, where *d* represents the particle size at a given time point and *d*_0_ indicates the initial size immediately after preparation. A *d*/*d*_0_ value of 1 suggests no size change, whereas values of >1 reflect aggregation. As presented in Figure 2d, the non-cross-linked BCNC-2 (negative control) exhibited a continuous increase in *d*/*d*_0_ throughout the incubation period, with particle size rising from 75 to 243 nm by day 3. In contrast, both BCNC-2 and BCNC-3 maintained *d*/*d*_0_ values close to 1 during the first 3 days. On day 3, their sizes remained nearly unchanged at 80 and 171 nm, compared with their initial sizes of 80 and 162 nm, respectively. However, by day 5, both formulations showed a considerable increase in *d*/*d*_0_, indicating the onset of aggregation. For example, BCNC-2 and BCNC-3 were measured to have *d*/*d*_0_ values of 6.3 and 3.6 after a 5-day incubation. Such a result suggested that both BCNC-2 and BCNC-3 maintain their particulate structure for up to 3 days in PBS (pH 7.4) but started to disassemble thereafter. To further evaluate colloidal stability, BCNC-3 was incubated under physiologically relevant conditions, including 1000-fold dilution (mimicking NPs suffering from bloodstream dilution), NaCl solution (0.1M), and an esterase-containing solution (1 mg/mL) (Figure S5). The *d*/*d*_0_ values for BCNC-in the three tested media on day 3 were measured as 1.8, 0.8, and 1.5 (after deducting the particle size contribution from esterase). These values indicate that BCNC-3 can retain its particulate structure in the test physiological environments for at least 3 days. To assess storage stability, BCNC-3 was incubated in PBS (pH 7.4) at 25 °C for 7 days. On day 7, the *d*/*d*_0_ value was recorded as 1.1, suggesting that the particle size remained constant during incubation. This result demonstrates the excellent storage stability of BCNC-3.

Moreover, CPLA-60 was converted into CPLA NCs via the same NC preparation procedure as for BCNC-3. CPLA NCs were subsequently employed as a non-PEGylated counterpart of BCNC-3. Both NCs were incubated in two physiologically mimicking media, including BSA (0.1%)-containing solution and NaCl solution (0.1 M), for 3 days, followed by monitoring their size change via DLS (Figure S6). After incubation in the BSA-containing solution, the *d*/*d*_0_ values for CPLA NCs and BCNC-3 on day 3 were measured as 1.8 and 1.2, respectively. When the incubation medium was changed to a NaCl solution, the *d*/*d*_0_ values for CPLA NCs and BCNC-3 on day 3 were 1.3 and 1.2, respectively. The DLS results showed that CPLA NCs exhibited significant particle aggregation under these incubation conditions. In contrast, BCNC-3 maintained the original particle size after being treated with the same incubation conditions as CPLC NCs. Despite having a similar particle core structure with CPLA NCs, BCNC-3 possesses an additional PEG shielding layer that protects against interactions with serum components such as BSA and NaCl.^59^ Accordingly, its particle structure was well preserved in the presence of these components, particularly compared to the non-PEGylated counterpart CPLA NCs.

Such colloidal stability results were presumably related to the hydrolytic degradation of the constituent polymers. In this context, the constituent polymers, including PEG-PLA-CPLA-80 and CPLA-60 for BCNC-3, were measured for their degradation rates when dissolved unimolecularly in different physiologically mimicking environments rather than in particulate form. The molecular weight changes were monitored by GPC (Figure S7). For PEG-PLA-CPLA-80, the *M*_i_/*M*_0_ values decreased to 0.2 and 0.3 at pH 7.4 and 5.5 PBS solutions, respectively, after 24 h (Figure S7a,c). Based on these values, PEG-PLA-CPLA-80 was considered to have undergone complete degradation of its PLA-based segments after 24 h incubation in the physiologically mimicked environments of the study based on the measured *M*_i_/*M*_0_ values. At the same time, the GPC curves continued to show peaks corresponding to the PEG segments at 35.7 mL after 24 h of incubation at the two study pH values (Figure S7a,c). This indicates that PEG is nondegradable in the test physiologically mimicking environments.^60^ However, the used PEG with a molecular weight lower than 10 kDa was reported to be readily eliminated from the human body.^60^ As for CPLA-60, it was also completely degraded at pH 7.4 (*M*_i_/*M*_0_ = 0.1) after 24 h of incubation, whereas only a small portion of polymer fragments remained at a pH 5.5 PBS solution (*M*_i_/*M*_0_ = 0.3) (Figure S7b,d). Overall, both PEG-PLA-CPLA-80 and CPLA-60 almost degraded completely after 24 h incubation at pH 7.4 and pH 5.5, whereas both degraded faster at pH 7.4 than at pH 5.5. Such degradation results indicate that the constituent components for BCNCs can degrade into small molecular weight species, ultimately being eliminated from the human body. This is because polymers with a molecular weight lower than 40 kDa were reported to be readily excreted from the human body.^61^

The half-life of free Dox in the human body was reported to be approximately 30–40 h.^62^ Moreover, the circulation time for most of the reported NPs in mice was observed to be shorter than 72 h.^63,64^ With a stable particulate morphology for at least 72 h, BCNCs are expected to minimize premature drug leakage during blood circulation or before reaching target tissues. Based on the degradation results, upon payload delivery, BCNCs are expected to degrade into small species that can be excreted from the body.^19^ Accordingly, the safety concerns associated with BCNCs can be substantially reduced. In addition to their well-defined capsular structure, BCNC-3 is characterized by enhanced colloidal stability, favorable particle size, tunable PEG segment density, and high zeta potential. Due to these biomedical advantages, BCNC-3 was selected as the NC material for coloading therapeutic drugs and genes to overcome MDR.

### In Vitro Drug and Gene Release from BCNCs

Using BCNC-3 as the carrier formulation, a hydrophilic gene Pgp-siRNA and a hydrophobic anticancer drug, Dox, were used as two chemically distinct model therapeutic agents (Scheme 1c). For Pgp-siRNA loading, prepared BCNC-3 was directly mixed with Pgp-siRNA for 40 min at various BCNC-3/Pgp-siRNA (NC/siRNA) weight ratios. The minimal NC/siRNA weight ratio required for complete Pgp-siRNA loading into BCNC-3 was determined by gel retardation assay. Upon the determination of the minimal NC/siRNA weight ratio, Dox-BCNC-3 was prepared by dissolving a specified amount of Dox in the nanoemulsion oil template prior to BCNC-3 formation. Subsequently, the resulting Dox-BCNC-3 was mixed with Pgp-siRNA for 40 min at the optimal NC/siRNA weight ratio, yielding Pgp-siRNA and Dox-coloaded BCNC-3 (Pgp-siRNA/Dox-BCNC-3), and the Dox loading efficiency was determined by UV–vis analysis.

An agarose gel retardation assay was first employed to evaluate the Pgp-siRNA loading capacity of BCNC-3. In the assay, BCNC-3 and Pgp-siRNA were mixed with different NC/siRNA weight ratios, resulting in Pgp-siRNA-BCNC-3 samples. The assay result showed that BCNC-3 fully retarded the Pgp-siRNA electrophoretic migration at a BCNC-3/Pgp-siRNA weight ratio of 16:1, indicating that BCNC-3 can fully load with Pgp-siRNA at an NC/siRNA weight ratio higher than 16:1 (Figure 3a). In contrast, BCNC-2, which has a higher PEG segment density than BCNC-3, was unable to completely retard Pgp-siRNA migration even at a BCNC-2/Pgp-siRNA weight ratio of 64:1 (data not shown). Such a result suggested that an ability to adjust the surface PEG density is crucial on the siRNA loading capacity of BCNCs. Previous reports also revealed that excessively high surface PEG density hampered gene adsorption on charged NPs.^54^ According to the assay result, the BCNC-3/Pgp-siRNA weight ratio for preparing Pgp-siRNA-BCNC-3 was set at 16:1, and the gene LC was calculated to be 6 wt %.

**Figure 3:**
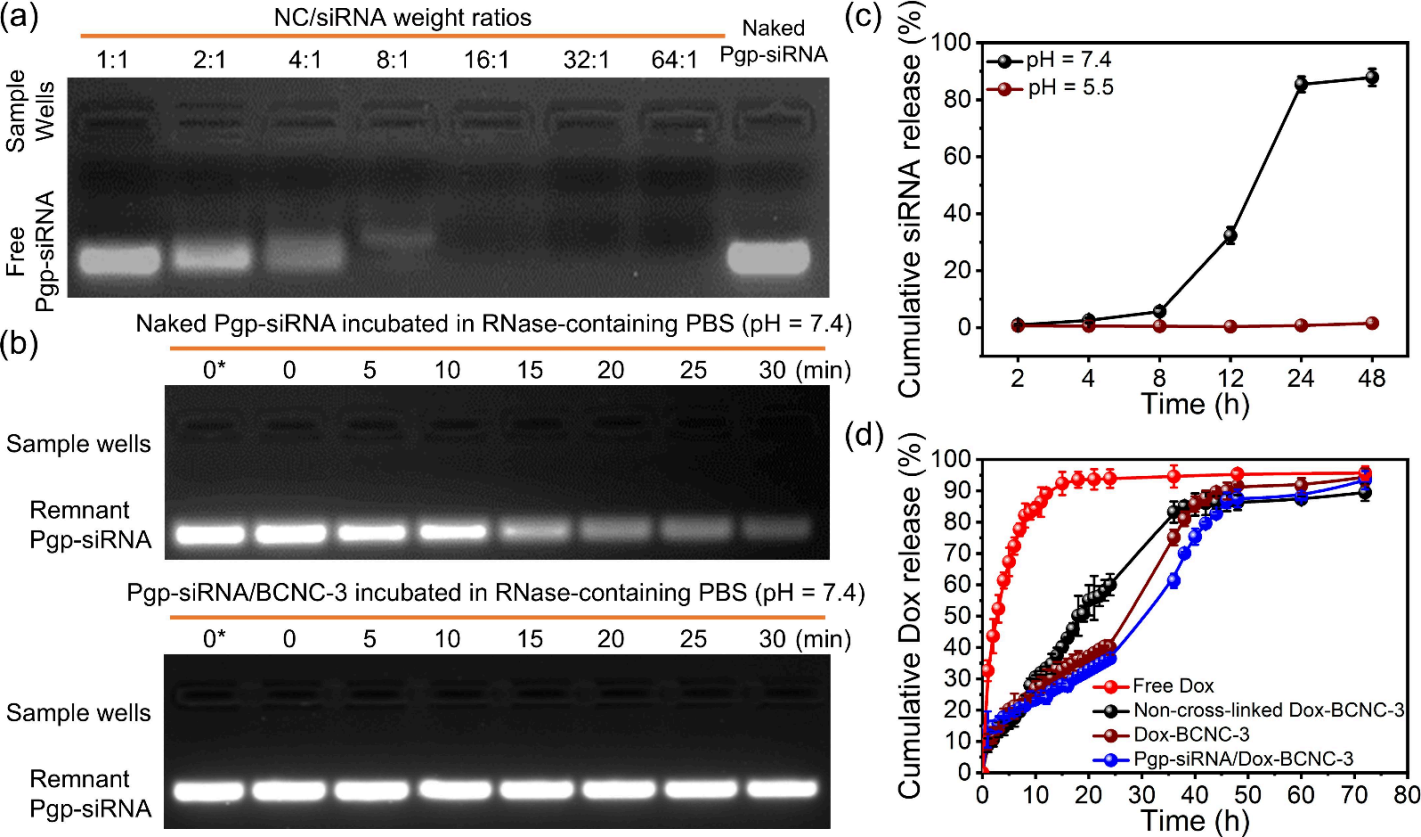
(a) Agarose gel electrophoresis results of Pgp-siRNA loaded in BCNC-3 at increasing NC/siRNA weight ratios. (b) Agarose gel electrophoresis results of naked Pgp-siRNA and Pgp-siRNA-BCNC-3 after incubation in a RNase-containing PBS solution (pH 7.4) for 5–30 min. After incubation, the siRNA molecules from the test samples were detached by SDS, followed by conducting the electrophoresis tests. For the trial of 0*, the electrophoresis result corresponds to unincubated intact siRNA. (c) siRNA release profiles from Pgp-siRNA-BCNC-3 at pH 7.4 and pH 5.5. (d) Dox release profiles from free Dox, non-cross-linked BCNC-3, Dox-BCNC-3, and Pgp-siRNA/Dox-BCNC-3 in a pH 7.4 PBS solution and 37 °C. Error bars represent standard deviations from three independent experiments.

Moreover, an enzymatic protection assay was employed to ensure the siRNA protection ability of BCNC-3 (Figure 3b). In the assay, naked Pgp-siRNA and Pgp-siRNA-BCNC-3 at an NC/siRNA weight ratio of 16:1 were incubated in a medium containing 0.25 μg/μL of RNase for 30 min. After the incubation and a detachment treatment, most naked Pgp-siRNA lost its electrophoretic band after 15 min, whereas Pgp-siRNA from Pgp-siRNA-BCNC-3 retained a clear bright band after 30 min of incubation. Using the band brightness of intact Pgp-siRNA as the reference, the residual siRNA after 30 min incubation in an RNase-containing PBS solution was 6.5 for naked siRNA and 84.0% for Pgp-siRNA-BCNC-3 (Figure S8). To further evaluate the anti-RNase effect of BCNC-3 on loaded Pgp-siRNA, the incubation time was extended from 30 min to 12 h (Figure S9). According to the band brightness, the residual siRNA of Pgp-siRNA-BCNC-3 was 80.9% of intact siRNA after 4 h of incubation, whereas the electrophoretic band of naked siRNA almost disappeared. After 12 h of incubation, the residual siRNA of Pgp-siRNA-BCNC-3 remained at 64.1% relative to intact siRNA. These results indicate that naked Pgp-siRNA were fully degraded by RNase after only 4 h, whereas BCNC-3 effectively protected the loaded Pgp-siRNA from RNase attack for at least 12 h.

Upon confirmation of the protection ability on Pgp-siRNA, Pgp-siRNA-BCNC-3 was evaluated for its Pgp-siRNA release profiles at pH 5.5 and pH 7.4 (Figure 3c). The Pgp-siRNA release percentages of Pgp-siRNA-BCNC-3 were calculated according to the electrophoretic band brightness (using the band brightness of naked Pgp-siRNA as the full siRNA release value) at different incubation times and pH values (Figure S10). Results showed that Pgp-siRNA-BCNC-3 exhibited a higher release rate and amount at pH 7.4 than at pH 5.5. At 12 and 24 h, 32.3 and 85.4% of the loaded Pgp-siRNA were released at pH 7.4, whereas only 0.4 and 0.8% were released at pH 5.5. This difference in Pgp-siRNA release amount is due to the different degradation rates of the constituent CPLA segment at the two pH values. Previously, we have demonstrated that CPLAs degraded more rapidly at pH 7.4 than at pH 5.5.^47^ Accordingly, BCNC-3 exhibited an enhanced Pgp-siRNA release amount at pH 7.4 compared to that at pH 5.5. Additionally, Pgp-siRNA-BCNC-3 showed a considerable amount of Pgp-siRNA release at pH 7.4 after 8 h. It was considered that the high degradation level of the CPLA segments from the constituent polymers of BCNC-3 after incubation in pH 7.4 PBS for 4 h (Figure S7) resulted in the enhanced Pgp-siRNA release as the adsorption and loading of Pgp-siRNA on BCNC-3 rely on electrostatic interaction of the positively charged CPLA segments and negatively charged Pgp-siRNA. Degradation of the CPLA segments disrupts these interactions, promoting the release of adsorbed Pgp-siRNA. Although pure CPLA segments started to have significant degradation after 4 h at pH 7.4, the Pgp-siRNA release amount remained lower than 5.6% before 8 h. BCNC-3 was measured to retain an intact particulate structure at pH 7.4 for up to 3 days (Figure S4). Such a particular structure is considered to hinder the release of the loaded Pgp-siRNA despite the constituent CPLA segments starting to decompose into small species. It is thought that when the CPLA segments of BCNC-3 degrade into a certain level, creating a critical void space within BCNC-3, the diffusion resistance for the loaded Pgp-siRNA is significantly reduced, subsequently resulting in an accelerated Pgp-siRNA release after 8 h, as presented in Figure 3c.^65^ In gene therapy, endosomal/lysosomal escape and the subsequent cytosolic unpacking of therapeutic genes are considered two major challenges for gene delivery vectors.^66^ Based on the release result, BCNC-3 is thought to reduce leakage of the loaded gene therapeutics in acidic endosomal/lysosomal environments while promoting the unpacking amount of the loaded gene cargos in the neutral cytosolic compartment.^49^ With its high stability under acidic conditions, BCNC-3 is considered to protect the loaded genes from decomposition during endosomal entrapment and lysosomal hydrolase attack, ultimately resulting in high endosomal escape efficiency for the loaded genes.^67^ Such an endosomal escape capability is thought to be an important factor affecting the transfection efficiency of BCNC-3.

Upon demonstrating the Pgp-siRNA loading capacity, BCNC-3 was employed to load with the hydrophobic anticancer drug Dox. Through the mentioned *in situ* Dox loading process, Dox-BCNC-3 was observed to have a drug LC of 8 wt %, which is a much higher value than that of conventional micellar systems.^68^ Dox-BCNC-3 was further loaded with Pgp-siRNA at an NC/siRNA weight ratio of 16:1 through the mentioned procedure, resulting in the preparation of Pgp-siRNA/Dox-BCNC-3. Moreover, the Dox release profile of Pgp-siRNA/Dox-BCNC-3 was measured in a pH 7.4 PBS solution, with those from free Dox, non-cross-linked Dox-BCNC-3, and Dox-BCNC-3 (without Pgp-siRNA loading) as control groups. As presented in Figure 3d, free Dox, non-cross-linked Dox-BCNC-3, Dox-BCNC-3, and Pgp-siRNA/Dox-BCNC-3 exhibited cumulative Dox release amounts of 72.4, 17.4, 21.2, and 19.6%, respectively, after a 6 h incubation period at 37 °C, suggesting that there was no appreciable burst release associated from the NCs, whereas free Dox displayed a significant burst release. Nonetheless, at 24 h, the cumulative release amounts of free Dox and non-cross-linked Dox-BCNC-3 were 93.9 and 60.1%, respectively, which were significantly higher than those of Dox-BCNC-3 and Pgp-siRNA/Dox-BCNC-3 (40.0 and 36.5%, respectively). This result implies that free Dox was almost released out the feeding drug amount from a dialysis bag after 24 h, accounting for the clinical limitations such as the short half-life time and the quick bloodstream elimination.^69^ Additionally, it was observed that non-cross-linked Dox-BCNC-3 and Dox-BCNC-3 (with cross-linkages) exhibited comparable Dox release amounts of 17.4 and 21.2% at 6 h. Meanwhile, after 24 h, non-cross-linked Dox-BCNC-3 exhibited a Dox release of 60.1%, which was much higher than the 40.0% observed for Dox-BCNC-3. These results indicate that non-cross-linked BCNC-3 can still retain the particulate structure at 6 h, thus displaying a drug release amount comparable to that of the cross-linked counterpart. After 24 h, the constituent polymers for non-cross-linked Dox-BCNC-3 began to have severe degradation (Figure S7). Without cross-linkages, it was considered that the cavity of non-cross-linked Dox-BCNC-3 became much leakier than that of Dox-BCNC-3 having cross-linkages, thereby resulting in a higher drug release amount than the cross-linked counterpart. Additionally, the adsorption of Pgp-siRNA on the NC surface was observed to reduce the level of Dox release from Dox-loaded BCNCs. At 24 and 36 h, Dox-BCNC-3 showed cumulative Dox release of 40.0 and 75.1%, whereas Pgp-siRNA/Dox-BCNC-3 exhibited 36.5 and 61.3%, respectively. Moreover, Pgp-siRNA/Dox-BCNC-3 displayed a sustained and slow Dox release between 6 and 24 h (from 19.6 to 36.5%), followed by an accelerated release between 24 and 48 h (from 36.5 to 87.6%). These results suggest that the integrity of the NC structure of Pgp-siRNA/Dox-BCNC-3 was presumably affected after 48 h. With this disintegration property, the NCs are expected to be ultimately eliminated from the human body after delivering the Dox and Pgp-siRNA payloads to target tissues.^70^ Moreover, a comparison of the release profiles of loaded Dox and Pgp-siRNA from BCNC-3 is presented in Figure S11. From this comparison, it was observed that after 8 h, Pgp-siRNA/Dox-BCNC-3 exhibited a faster release of Pgp-siRNA than did Dox, whereas Dox release was much slower than that of free Dox. Before 8 h, the release of both Pgp-siRNA and Dox was dominated by diffusion.^71^ The Pgp-siRNA diffusion from BCNC-3 was strongly limited by the cationic shell layer of BCNC-3 via an electrostatic interaction at the early release stage. In contrast, the Dox diffusion was not significantly affected by the cationic shell layer due to the hydrophobic property of Dox. As a result, Dox was released faster than Pgp-siRNA from Pgp-siRNA/Dox-BCNC-3 before 8 h. However, CPLA segments, the major cationic resource of BCNC-3, degraded significantly after 8 h (Figure S7). Due to the degradation, the Pgp-siRNA release was considerably enhanced because the electrostatic interaction with BCNC-3 had been significantly reduced. Accordingly, the loaded Pgp-siRNA from Pgp-siRNA/Dox-BCNC-3 exhibited a faster release rate than the coloaded Dox in the late release period. Previously, Yan and co-workers designed a chitosan-based micelle for coloading Dox and Bcl-2 siRNA for overcoming MDR in hepatoma cells,^72^ in which 90.2% of Dox and 81.3% of Bcl-2 siRNA were released within 10 h. However, Pgp-siRNA/Dox-BCNC-3 showed Dox and Pgp-siRNA cumulative release amounts of 36.5 and 85.4%, respectively, in 24 h. The higher release amount of Pgp-siRNA relative to that of Dox enables Pgp-siRNA/Dox-BCNC-3 to silence Pgp expression in advance, followed by releasing loaded Dox to kill the sensitized cancer cells. Therefore, in addition to its coloading capability for hydrophobic anticancer drugs and hydrophilic gene therapeutics, the sequential release behavior of Pgp-siRNA/Dox-BCNC-3 can benefit the therapeutic efficacy of the loaded anticancer drugs in MDR cancer cells.

### In Vitro Cellular Uptake and Endolysosomal Escape of BCNCs

To ensure the biosafety of BCNCs, BCNC-3 was used as a representative NC to measure the cytotoxicity via an MTT assay. Four different cell lines, including MCF-7/ADR, MCF-7, HepG2, and HeLa cells, were used as the test cells, which were treated with BCNC-3 at concentrations ranging from 10 to 640 μg/mL for 48 h. Using the untreated test cells as the control (i.e., 100% cell viability), BCNC-3 at all the incubation concentrations showed viability values higher than 85% (Figure S12), indicating that BCNC-3 had insignificant cytotoxicity and can be used as a safe carrier for drug and gene delivery applications.

Sequentially, CLSM was employed to observe the cell uptake of Dox (red fluorescence) and siFAM (green fluorescence) by MCF-7/ADR cells, an MDR cell line, with the use of BCNC-3 as the carrier (Figure 4a). Additionally, the nucleus was stained with DAPI (blue fluorescence) to evaluate whether the therapeutics accumulated in the cytoplasm of treated cells.

**Figure 4:**
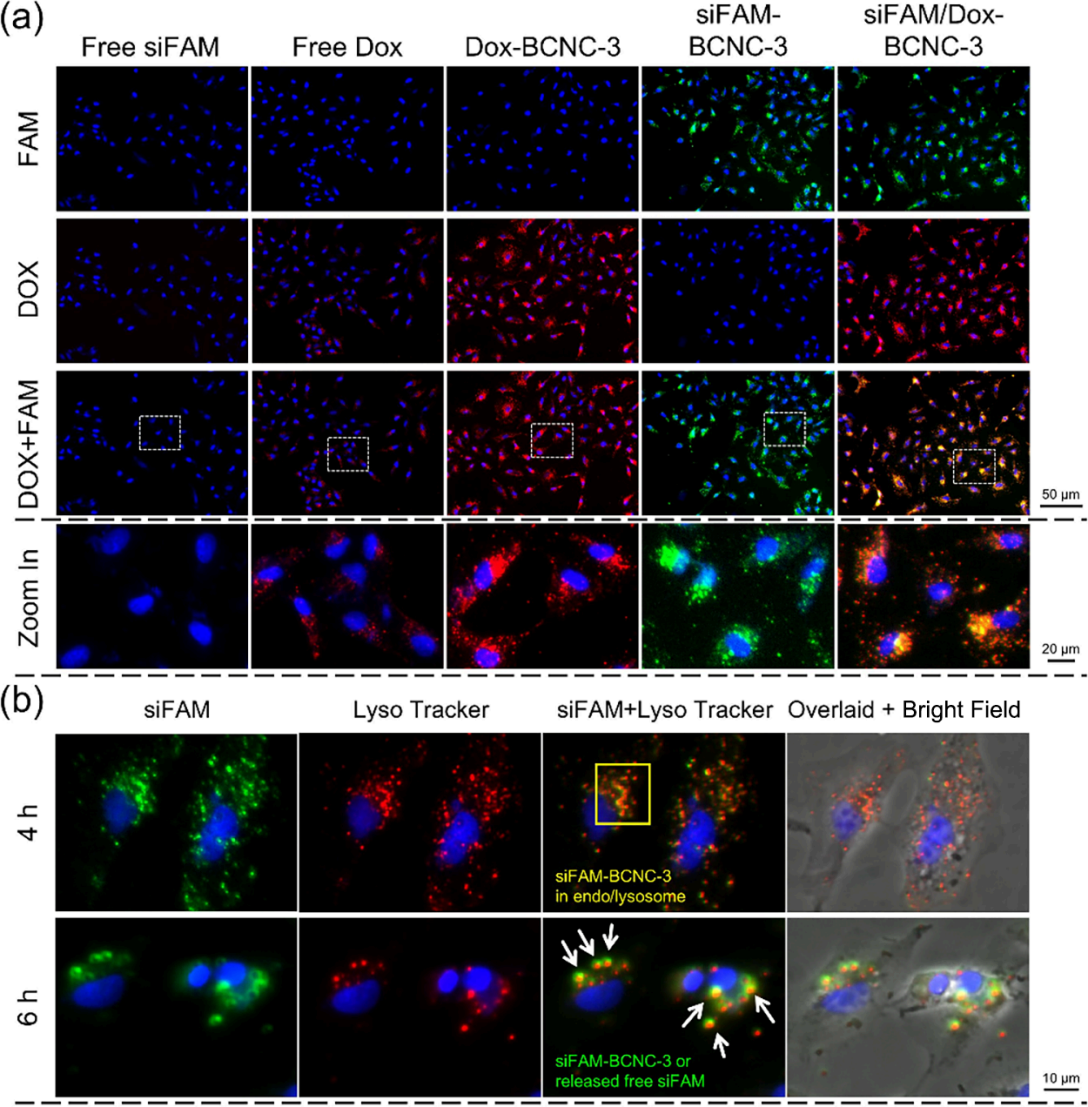
(a) Fluorescence microscopy images of MCF-7/ADR cells treated with free siFAM, free Dox, Dox-BCNC-3, siFAM-BCNC-3, and siFAM/Dox-BCNC-3 after incubation in a cell culture medium for 4 h. In the fluorescence images, blue, red, and green fluorescence signals represent the cell nucleus, Dox, and siFAM, respectively; scale bar = 50/20 μm. (b) Endolysosome escape and colocalization analysis of siFAM-BCNC-3 after incubation in a cell culture medium for 4 and 6 h. In the analysis, blue, red, and green fluorescence signals represent the cell nucleus, endosomes or lysosomes, and siFAM; scale bar = 10 μm.

The observation time was set to 4 h based on previous reports showing that NPs typically require 4–6 h to distribute throughout the cytoplasm.^73^

It was observed that free siFAM was hardly taken up by MCF-7/ADR cells, while free Dox exhibited only limited cellular uptake in these cells. The difficulty of MCF-7/ADR cells in directly taking up free siFAM can be ascribed to two major reasons. First, free siRNA itself exhibits a strong negative charge, resulting in a strong electrostatic repulsion between the siRNA phosphate backbones and the negatively charged cell membranes.^74^ This repulsion is considered an important factor hindering cellular uptake. Additionally, free siRNA is readily degraded by extracellular nucleases.^75^ The susceptibility of free siRNA to enzymatic degradation also makes it difficult for cells to take up. These results indicate that, without the protection of gene delivery vectors, naked siRNA is readily degraded in a cell culture environment,^76^ ultimately resulting in poor cellular uptake. Although Dox can enter cells via diffusion,^77^ its accumulation was very limited, according to the low red fluorescence intensity. This limited uptake is presumably due to the Pgp efflux effect in MCF-7/ADR cells.^78^ In contrast, when BCNC-3 was used as the carrier, the cellular uptake of both Dox (Dox-BCNC-3) and siFAM (siFAM-BCNC-3) was significantly increased in MCF-7/ADR cells according to the red or green fluorescence intensity. These results revealed that BCNC-3 is capable of protecting the bioactivity of siRNA and promoting the cell uptake amount via an endocytosis mechanism.^49^ It was considered that BCNC-3 can effectively protect siFAM from nuclease attack, thus retaining the molecular integrity and subsequent bioactivity. In addition, BCNC-3 provides the loaded siFAM with a positively charged property, which was considered to be a crucial factor facilitating the cell uptake in MCF-7/ADR cells. Moreover, BCNC-3 can promote the Dox uptake in a MDR cell through an evading effect, which prevents Pgp on the cell membrane from recognizing the encapsulated Dox, subsequently allowing more Dox to be accumulated in the cells.^79^ Additionally, for the trial of siFAM/Dox-coloaded BCNC-3 (siFAM/Dox-BCNC-3), the treated MCF-7/ADR cells exhibited strong red and green fluorescence intensities around the nucleus, indicating that most of the loaded hydrophobic anticancer drugs and hydrophilic siRNA genes have been simultaneously delivered into the cells. In addition, the observed yellow fluorescence resulting from the colocalization of the signals from Dox and siFAM suggests that BCNC-3 can simultaneously deliver these two chemically distinct therapeutics into a MDR cell line. Taken together, it is considered that the positively charged surface and NC structure facilitate the uptake of BCNC-3 by MDR cells. Having a negatively charged surface, cancer cells therefore exhibit a high affinity for positively charged BCNC-3.^80^ Additionally, the tunable surface PEG density alleviates the endocytosis difficulty of BCNC-3.^54^ With an NC structure, BCNC-3 is unable to be recognized by the Pgp around the surface of MCF-7/ADR cells.^81^ Accordingly, the loaded Dox has been massively delivered into the cells with minimal concern of being pumped out by Pgp. Moreover, the loaded siFAM will be replaced by Pgp-siRNA to further observe its effect on enhancing the therapeutic efficacy of Dox through knockdown.

The entrapment of internalized siRNA is considered a major factor affecting the RNA interference (RNAi) mechanism and the resulting transfection efficiency. This is because siRNA is readily degraded in endolysosomal compartments due to lysosomal enzymes and the acidic environment.^82^ To efficiently perform the RNAi process, internalized siRNA must escape from the endosome and lysosome within a short time to avoid degradation. In this context, siFAM-BCNC-3 was prepared, and the nucleus and lysosomes of MCF-7/ADR cells were stained using DAPI and LysoTracker Red (red fluorescence; Figure 4b). CLSM results showed that after 4 h of incubation, a colocalized yellow fluorescence signal, resulting from the overlap of the green fluorescence from siFAM and the red fluorescence of the lysosomes, was observed and highlighted with a yellow frame in Figure 4b. The yellow fluorescence indicates that most of the delivered siRNA remained within endolysosomal vesicles. After 6 h of incubation, as indicated by the arrows in Figure 4b, distinct green (from siFAM) and red (from stained lysosomes) fluorescence signals were clearly observed. This fluorescence separation indicates that the loaded siFAM from siFAM-BCNC-3 successfully escaped from endolysosomal compartments through BCNC-3.^83,84^ To further corroborate this observation, the colocalization of the green and red fluorescence signals was quantified using ImageJ (coloc2 plugin) based on the calculated Pearson’s correlation coefficients from the two observed incubation times (Figure S13).^49^ The coefficients were 0.8 and 0.64 at 4 and 6 h, respectively. The reduced coefficient value indicates a decreased overlap between the two fluorescence signals, further supporting the endolysosomal escape ability of BCNC-3. These results demonstrate that BCNC-3 can facilitate the escape of loaded genes from endo/lysosomal compartments, thereby enabling their transfection functions in the cytoplasm.^85^ Consisting of PEG-PLA-CPLA-80/CPLA-60, BCNC-3 contains a considerable amount of tertiary amine due to the cationic side groups from the CPLA segments. It was reported that gene delivery vectors with tertiary side groups can facilitate endosomal escape through osmotic swelling and the proton sponge effect. This mechanism accounts for the endo/lysosomal escape ability of BCNC-3 and enables BCNC-3 to be used as a promising gene delivery platform for a variety of siRNA-based gene therapies.^48^

### Pgp Silencing Effect and In Vitro Anticancer Activity of BCNCs on MDR Cells

Upon confirmation of the siRNA and anticancer drug delivery capacities of BCNC-3, the Pgp silencing efficacy and in vitro anticancer activity against MDR cells were further studied. In this context, Pgp-siRNA/Dox-BCNC-3 and the corresponding experimental trials were incubated in MCF-7/ADR cells for 48 h. After the incubation, Pgp mRNA levels in the treated cells were measured through qRT-PCR. The results showed that Pgp mRNA levels in cells treated with free Pgp-siRNA, free Dox, and BCNC-3 ranged from 86.6 to 98.9% relative to the untreated MCF-7/ADR cells (Figure 5a). This indicates that the naked gene, small-molecule therapeutic agents, and NCs had a negligible effect on inhibiting the Pgp mRNA expression. It was additionally noted that the Dox-BCNC-3-treated cells exhibited a Pgp mRNA expression level of 74.4%. The reduced Pgp mRNA expression is presumably attributable to two mechanisms.^7^ First, due to its high interaction affinity with MCF-7/ADR cells, BCNC-3 may alter the composition of the cell membrane, thereby interfering with the activity of Pgp. Second, the evasion effect provided by BCNC-3 may impair Pgp recognition and subsequently reduce its catalytic activity.

**Figure 5:**
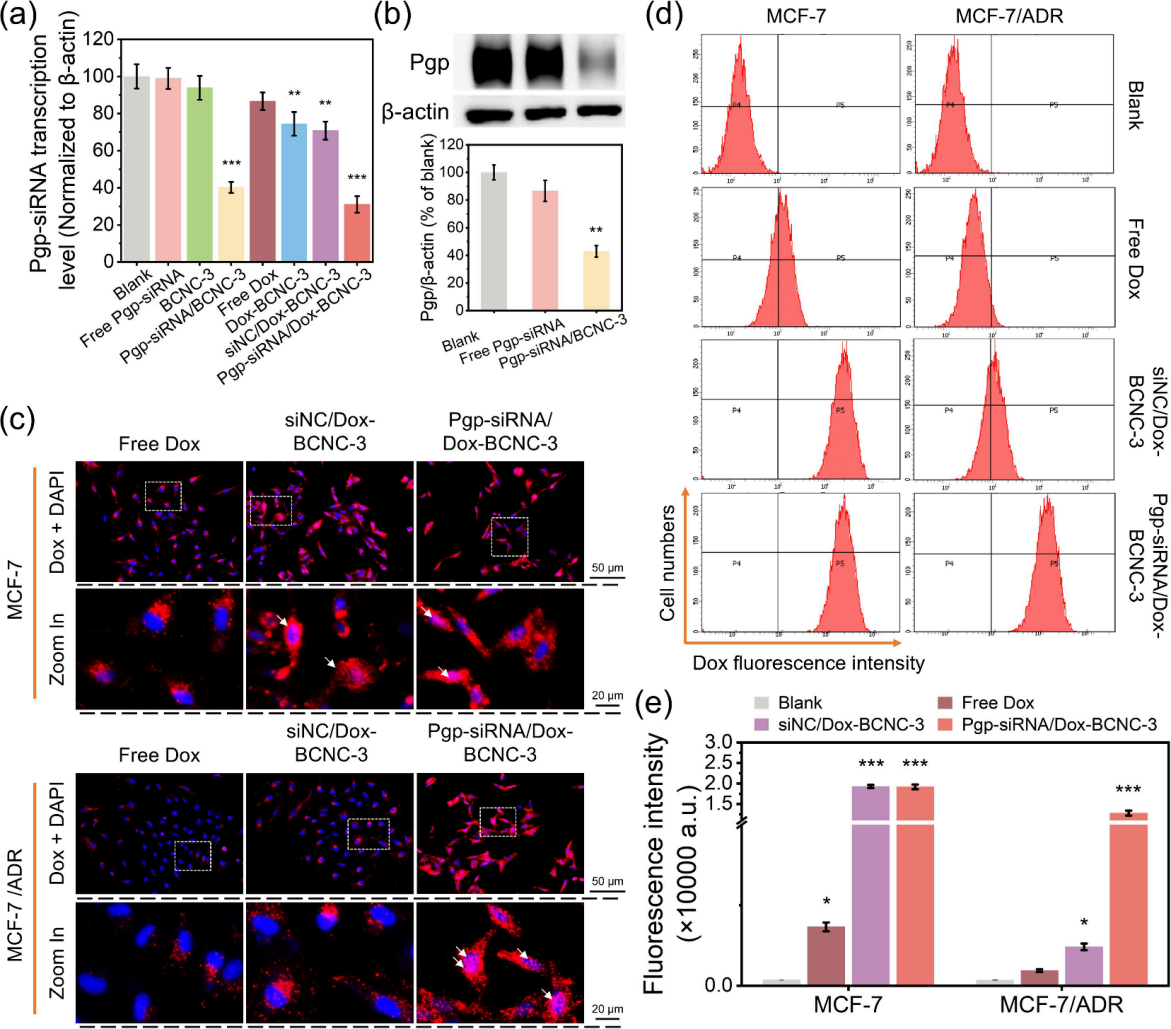
(a) Pgp mRNA expression levels (%) relative to the blank trial for free Pgp-siRNA, BCNC-3, Pgp-siRNA/BCNC-3, free Dox, Dox-BCNC-3, siNC/Dox-BCNC-3, and Pgp-siRNA/Dox-BCNC-3, measured by qPCR after 48 h of incubation in MCF-7/ADR cells. (b) Western blot analysis of Pgp expression in MCF-7/ADR cells treated with blank (untreated), free Pgp-siRNA, and Pgp-siRNA/BCNC-3 for 48 h. Using the band darkness from β-actin as the reference, quantitative Pgp expression levels were obtained by dividing the band darkness intensities from the trials to that from blank. (c) Fluorescence microscopy images of MCF-7 and MCF-7/ADR cells treated with free Dox, siNC/Dox-BCNC-3, and Pgp-siRNA/Dox-BCNC-3 after 24 h incubation; scale bar = 50/20 μm. (d) Cellular uptake results of free Dox, siNC/Dox-BCNC-3, and Pgp-siRNA/Dox-BCNC-3 in MCF-7 and MCF-7/ADR cells measured by flow cytometry after 24 h incubation. (e) Fluorescence intensity analysis based on cellular uptake results. Error bars represent standard deviations resulting from three independent experiments. The significance values were obtained by comparing the study trials to the blank group. *P < 0.05; **P < 0.01; ***P < 0.001.

In contrast, both the Pgp-siRNA-BCNC-3- and Pgp-siRNA/Dox-BCNC-3-treated cells exhibited a significantly decreased Pgp mRNA expression level, measured at 40.2 and 31.0%, respectively. At the same time, siNC/Dox-BCNC-3-treated cells displayed a Pgp mRNA expression level of 70.8%. These results confirmed that the major decrease in Pgp mRNA expression is due to the loaded Pgp-siRNA within BCNC-3. Moreover, BCNC-3 plays a vital role in protecting and delivering loaded Pgp-siRNA into MCF-7/ADR cells, thereby facilitating the RNAi pathway and sequentially reducing Pgp expression. In addition to qRT-PCR, Western blotting was performed on MCF-7/ADR cells (Figure 5b). Upon treatment with free Pgp-siRNA and Pgp-siRNA/BCNC-3, the Pgp expression levels in the treated MCF-7/ADR cells were downregulated to 86.7 and 42.9%, respectively, compared to the blank control. The Western blotting result for Pgp-siRNA-BCNC-3 was consistent with the qRT-PCR findings. Moreover, the Pgp silencing of BCNC-3 was also investigated in another MDR cell. As presented in Figure S14, Western blotting results showed that the Pgp expression levels of free Pgp-siRNA- and Pgp-siRNA/BCNC-3-treated MES-SA/Dx5 cells were measured to be 85.9 and 4.1%, respectively, relative to the blank control. These results indicate that BCNC-3-mediated delivery of Pgp-siRNA considerably downregulated Pgp expression in MES-SA/Dx5 cells. Taken together, it was considered that BCNC-3 can be used as a promising Pgp-siRNA delivery agent for down-regulating the Pgp expression in various MDR cancer cells.

Sequentially, BCNC-3-mediated Dox cell uptake in MCF-7 and MCF-7/ADR cells was observed through CLSM, and the observation time was set at 24 h. This is because our in vitro Dox release study showed that BCNC-3 with the loading of Dox exhibited a considerable release amount after 24 h of incubation in a pH 7.4 PBS solution. CLSM results showed that siNC/Dox-BCNC-3- and Pgp-siRNA/Dox-BCNC-3-treated MCF-7 cells exhibited significantly higher Dox fluorescence intensity than cells treated with free Dox-treated MCF-7 after 24 h of incubation. Meanwhile, MCF-7 cells treated with siNC/Dox-BCNC-3 and Pgp-siRNA/Dox-BCNC-3 had a comparable Dox fluorescence intensity (Figure 5c). As for the trial of free Dox, MCF-7 cells can only take up Dox by a diffusion mechanism, indicating that there is a Dox concentration equilibrium between cells and the cultivated medium.^86^ In contrast to free Dox, Dox-loaded BCNCs, such as siNC/Dox-BCNC-3 and Pgp-siRNA/Dox-BCNC-3, facilitated greater internalization of Dox by MCF-7 cells due to the electrostatic interaction between the positively charged BCNC-3 and the negatively charged MCF-7 cells.^87^ Additionally, the size of BCNC-3, which is below 200 nm, benefits the loaded Dox to be taken up through an endocytosis mechanism.^88^ In addition, the loaded Pgp-siRNA had no significant effect on the Dox uptake amount because MCF-7 cells are not a strong MDR cell.^89^ When the study cells changed to MCF-7/ADR cells, it was observed that after 24 h of incubation, Dox-treated MCF-7/ADR cells showed a very limited fluorescence intensity, which is much weaker than that from Dox-treated MCF-7 cells. Relative to Dox-treated MCF-7/ADR cells, siNC/Dox-BCNC-3-treated MCF-7/ADR cells exhibited a slight increase in the Dox fluorescence intensity. However, Pgp-siRNA/Dox-BCNC-3-treated MCF-7/ADR cells showed a very strong Dox fluorescence intensity. Such results suggested that Pgp on the surface and nucleus of MCF-7/ADR cells can recognize Dox, followed by pumping it out from the cells. Accordingly, the amount of Dox from free Dox is lower in MCF-7/ADR cells than that in MCF-7 cells. Moreover, although having an evading effect on the surface Pgp, siNC/Dox-BCNC-3 had only a slight improvement on the Dox cell uptake amount in MCF-7/ADR cells. This is because, after 24 h of incubation, approximately 36.5% of the loaded Dox (according to the release profiles) has been released from the capsular structure. The released Dox was subsequently recognized by the Pgp on the nucleus, followed by activating the pumping out mechanism, leading to poor Dox uptake.^90^ As compared to siNC/Dox-BCNC-3, Pgp-siRNA/Dox-BCNC-3 exhibited a considerably increased Dox cell uptake in MCF-7/ADR cells. By silencing Pgp, Pgp-siRNA/Dox-BCNC-3 can inhibit the function of Pgp in MCF-7/ADR cells, both at the cell membrane and the nuclear surface, thereby sensitizing the cells and alleviating MDR.^24^ Consequently, the Dox accumulation in these MDR cells is significantly increased due to the carrier functions of BCNC-3.

To confirm the CLSM results, flow cytometry was employed to measure the Dox fluorescence of MCF-7 and MCF-7/ADR cells, which were treated with free Dox, siNC/Dox-BCNC-3, and Pgp-siRNA/Dox-BCNC-3 (Figure 5d,e). In the flow cytometry analysis, the cell uptake of Pgp-siRNA/Dox-BCNC-3 in MCF-7 cells over time was first analyzed by flow cytometry to ensure an optimal incubation time (Figure S15). Flow cytometry results revealed that the Dox fluorescence intensity was increased by increasing incubation time, and the treated cells showed the maximal Dox intensity at 24 h. This indicates that MCF-7 cells had a saturated uptake level for BCNC-3 at 24 h. Accordingly, the incubation time to observe the Dox cellular uptake amounts of the test trials in MCF-7 and MCF-7/ADR cells was set to 24 h. In MCF-7 cells, results showed that siNC/Dox-BCNC-3 and Pgp-siRNA/Dox-BCNC-3 had a comparable Dox intensity, which is significantly higher than that from free Dox. In MCF-7/ADR cells, the Dox fluorescence intensity followed the order: Pgp-siRNA/Dox-BCNC-3 > siNC/Dox-BCNC-3 > free Dox. The flow cytometry results were in accordance with the CLSM results. Both results verified that the NC structure of BCNCs enhances the cellular accumulation of loaded hydrophobic anticancer drugs, even in cancer cells without MDR. Moreover, due to the capacity of coloading drugs and genes, BCNCs with a proper drug/gene therapeutic strategy can enhance the delivery of drugs such as Dox, into MDR cancer cells, for example, MCF-7/ADR cells. Owing to their material properties, BCNCs have been demonstrated to play a vital role in drug formulations designed to overcome MDR in cancer cells.

Sequentially, the anticancer efficacy of drug- and gene-loaded BCNCs was demonstrated on MCF-7 and MCF-7/ADR cells through an MTT assay (Figure 6). The incubation times were set as 48 and 72 h, respectively. This is because previous reports showed that Pgp-siRNA delivery by vectors exhibited a maximum gene knockdown effect at 48 h, and the effect remained for at least 96 h.^91^ After 48 and 72 h incubation, results showed that BCNC-3, Pgp-siRNA, and Pgp-siRNA-BCNC-3 exhibited negligible cytotoxicity under the NC incubation concentration of 6.25 μg/mL in MCF-7 and MCF-7/ADR cells. In MCF-7 cells, free Dox exhibited an antitumor efficiency (the percentage of cancer cells killed relative to the original cell number) of 19.2%, whereas Dox-BCNC-3 showed an efficiency of 48.3% at a Dox concentration of 0.5 μg/mL after 72 h of incubation, indicating that the enhanced cell uptake via the NC can promote the antitumor activity of the loaded Dox. An additional loading of siNC or Pgp-siRNA had no significant influence on the antitumor efficacy of Dox-BCNC-3. In MCF-7/ADR cells, compared to the antitumor efficacy of free Dox (5.2%), that of Dox-BCNC-3 was moderately increased to 18.2%, suggesting that the NC evading effect on overcoming MDR is limited. In contrast, the antitumor efficacy of Pgp-siRNA/Dox-BCNC-3 reached 55.6%, which is much higher than that of free Dox and Dox-BCNC-3, revealing that the loaded Pgp-siRNA successfully inhibits the Pgp expression, facilitates the Dox uptake, and ultimately enhances the cell killing efficiency on a MDR cancer cell. Notably, when the Pgp-siRNA was replaced with siNC, the resulting siNC/Dox-BCNC-3 only exhibited an antitumor efficiency of 16.2%, which is comparable to that of Dox-BCNC-3. As a result, the enhanced antitumor efficacy of Pgp-siRNA/Dox-BCNC-3 relative to that of Dox-BCNC-3 is due to the Pgp silencing function of loaded Pgp-siRNA.

**Figure 6:**
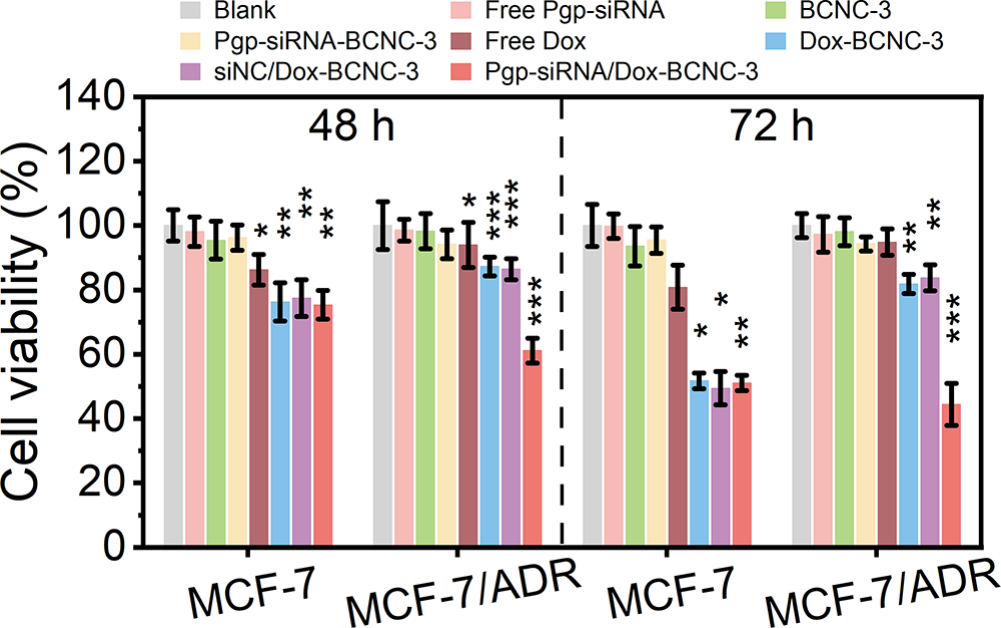
Cell viability of MCF-7 and MCF-7/ADR cells treated with free Pgp-siRNA, BCNC-3, Pgp-siRNA-BCNC-3, free Dox, Dox-BCNC-3, siNC/Dox-BCNC-3, and Pgp-siRNA/Dox-BCNC-3 after 48 and 72 h incubation, as measured by an MTT assay. The concentrations of BCNCs, Pgp-siRNA/siNC, and Dox were 6.25, 0.4, and 0.5 μg/mL, respectively. Error bars represent standard deviations from three independent experiments. The significance values were obtained by comparing the study trials to the blank group. *P < 0.05; **P < 0.01; ***P < 0.001.

Moreover, the antitumor efficiencies of therapeutic agent-loaded BCNC-3, such as Dox-BCNC-3, siNC/Dox-BCNC-3, and Pgp-siRNA/Dox-BCNC-3, were evaluated on MES-SA/Dx5 cells, followed by comparison with that of free Dox (Figure S16). Similar to the results from MES-SA/Dx5 cells at 72 h, Pgp-siRNA/Dox-BCNC-3 showed the antitumor efficiency of 43.4%, which was higher than 22.0% for free Dox, 19.1% for Dox-BCNC-3, and 22.9% for siNC/Dox-BCNC-3. These results indicate that the Pgp expression associated with MES-SA/Dx5 cells can be effectively inhibited by the Pgp-siRNA delivered by BCNC-3. Due to the inhibition, the loaded Dox achieved significantly enhanced antitumor efficiency compared with free Dox.

### Preliminary In Vivo Anticancer Activity Study of BCNC-Mediated Therapy

A preliminary in vivo study for evaluating the biodistribution, cardiotoxicity, and antitumor effect of BCNC-3-mediated therapeutic formulations has also been performed (Figures 7, 8, and 9). The biodistribution of BCNC-3 was investigated in MCF-7-bearing mice using hydrophobic Dir as a fluorescence probe with free Dir as the control (Figure 7a). As for free Dir, no significant fluorescence intensity was found at the tumor site at the observation times. In contrast, a detectable fluorescence signal was observed at the tumor site (area marked by a red circle line) 12 h postinjection of Dir-BCNC-3, and it reached a maximum intensity at 48 h, followed by observing a decreased intensity at 72 h. These results indicate that Dir, used to mimic hydrophobic Dox, can be delivered into tumor tissues via BCNC-3 through improving the water solubility and rapid clearance of the loaded Dir and taking advantage of the enhanced permeability and retention (EPR) effect.^92,93^ It was also noted that significant fluorescence intensities were also observed in the liver at 12, 24, and 48 h, and the intensity started to decrease at 72 h. Such high accumulation is presumably due to the small particle size (<200 nm) and the positively charged surface.^93^ The decrease in the intensity also suggests that BCNC-3 started degrading into small molecular weight species, which were subsequently excreted from the mice. At 72 h postinjection of free Dir and Dir-BCNC-3, the injected mice were sacrificed and the Dir fluorescence intensities in the major organs were measured (Figure 7b,c). As compared with free Dir, Dir-BCNC-3 exhibited a 2.4-fold higher fluorescence intensity at the tumor tissues, demonstrating that the mentioned carrier functions are able to facilitate the tumor site accumulation for the loaded agents. However, substantial Dir accumulation was also observed in the liver and spleen for Dir-BCNC-3. This is presumably because the mononuclear phagocyte system (MPS), consisting of Kupffer cells in the liver and macrophages in the spleen, has taken up a considerable proportion of BCNC-3, subsequently affecting its accumulation in the tumor.^94^ In addition, the lack of targeting ligands such as RGD peptides, folic acid, transferrin, or aptamers of BCNC-3 was considered to limit the binding ability to tumor cell receptors.^95^ Moreover, the surface charge strength was selected based on the colloidal results, instead of the in vivo results. This may also influence the in vivo performances.^96^ To address the tumor accumulation issue, improvement in preventing BCNC-3 from recognition by MPS is of high importance in increasing the drug delivery efficiency of BCNC-3. Moreover, the decoration of a proper targeting moiety and the optimization regarding the surface charge strength were considered to enhance the tumor cell binding ability and to reduce the nonspecific interactions with serum proteins.^97^ Through the proposed strategies, resulting BCNC-3 is expected to exhibit an improved tumor accumulation capability.

**Figure 7:**
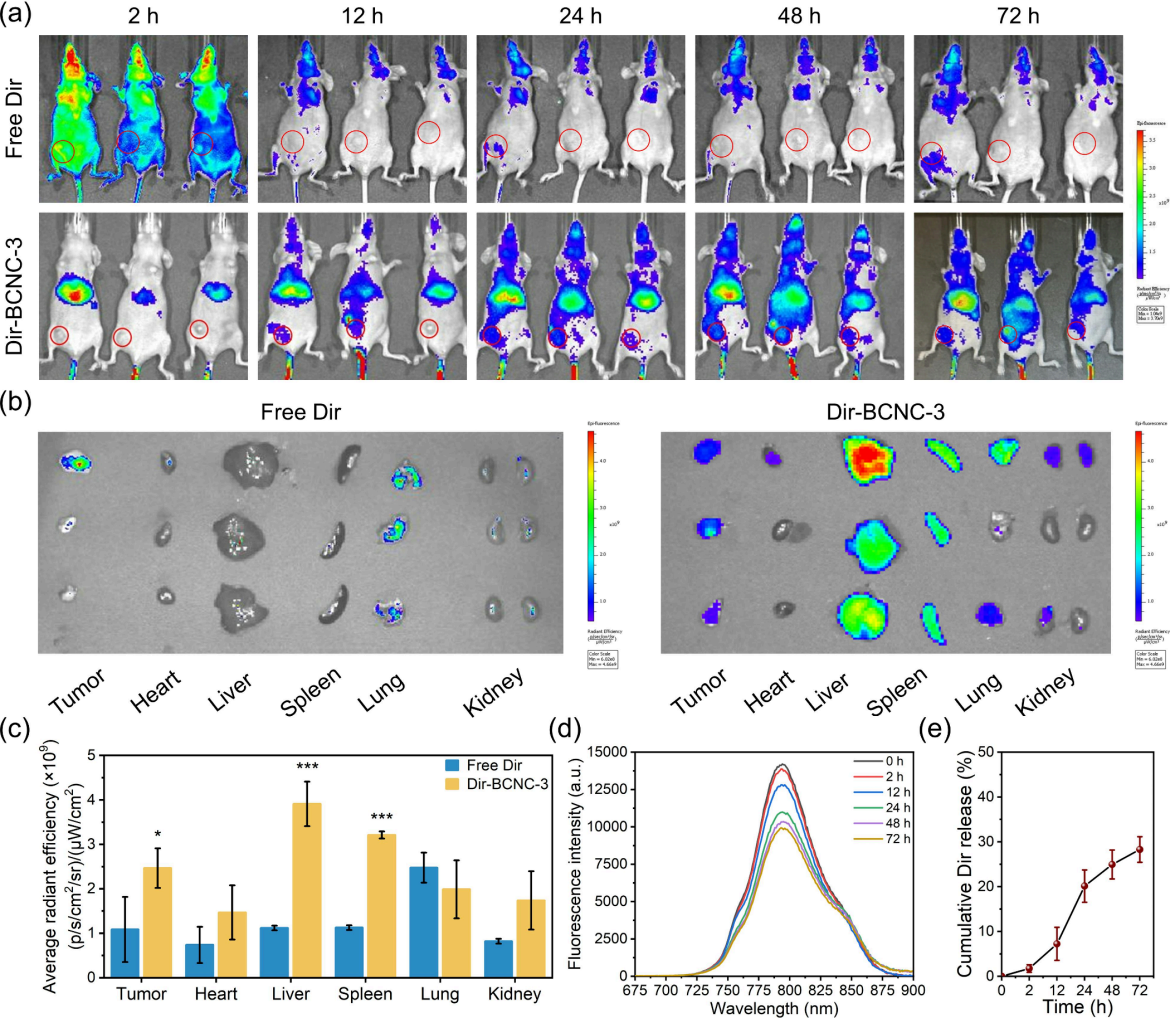
(a) In vivo fluorescence imaging of MCF-7 xenograft tumor-bearing mice at increasing time intervals following intravenous injection of free Dir and Dir-BCNC-3. (b) Ex vivo fluorescence imaging and (c) quantification fluorescence intensities of tumors and major organs excised from MCF-7 xenograft tumor-bearing mice 72 h postinjection of free Dir and Dir-BCNC-3. (d) Fluorescence emission spectra of Dir-BCNC-3 over a 72 h incubation period in PBS buffer (pH 7.4, 37 °C), under excitation at λ_ex_ = 754 nm. (e) Cumulative Dir release profile from Dir-BCNC-3 in PBS buffer (pH 7.4, 37 °C). Data are presented as mean ± SD (*n* = 3). The significance values were obtained by comparing the study trials to the free Dir group. *P < 0.05; **P < 0.01; ***P < 0.001.

**Figure 8:**
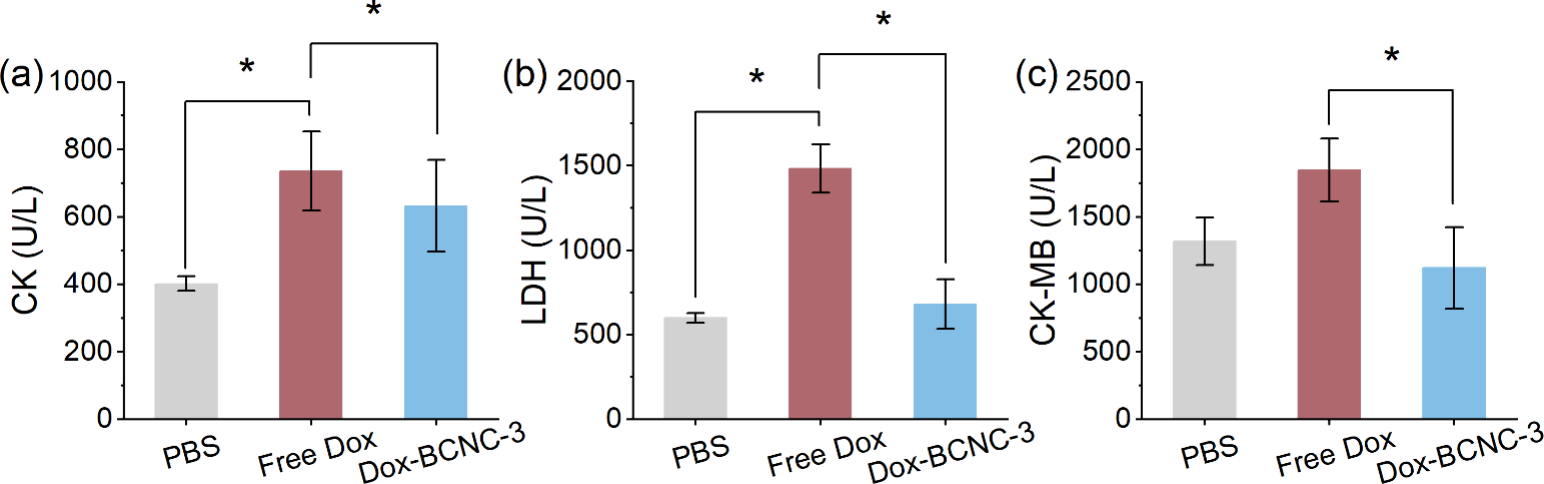
Serum biochemical indices of mice treated by intravenous injection with PBS, free Dox, and Dox-BCNC-3 at an equivalent Dox dose of 4 mg/kg after 14 days, with a total of six administrations. Each group included five mice 5 (*n* = 5). *P < 0.05.

**Figure 9:**
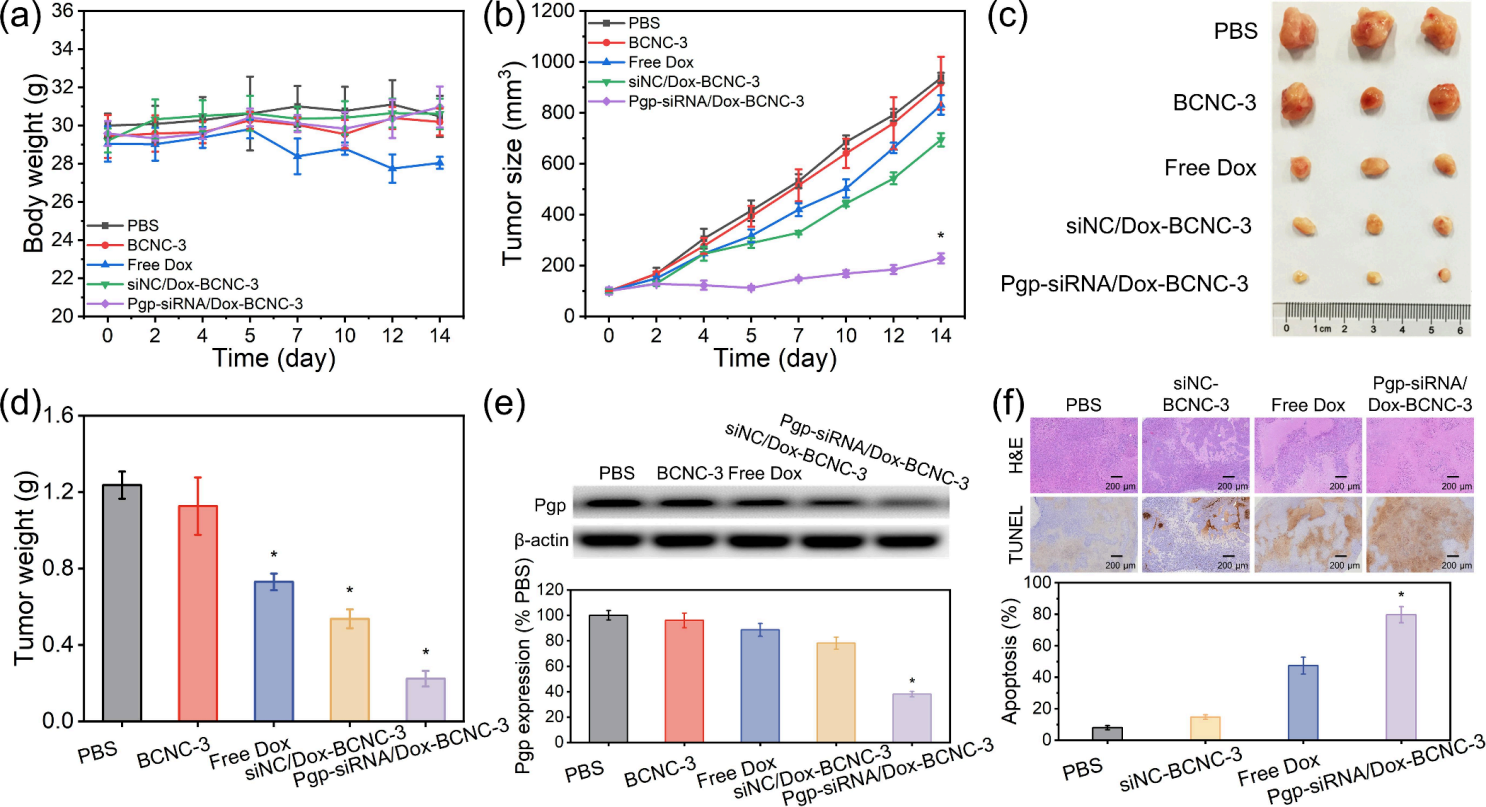
In vivo antitumor efficiency of different formulations against MCF-7/ADR tumors. Monitoring of (a) body weight change and (b) tumor size change in MCF-7/ADR xenograft tumor-bearing mice after treatment with PBS, BCNC-3, free Dox, siNC/Dox-BCNC-3, and Pgp-siRNA/Dox-BCNC-3. For each intravenous injection, the dose of siRNA and Dox were 40 and 68.4 μg, respectively. A total of intravenous injections were administered over 14 days. (c) Representative images and (d) tumor weights of excised tumors after six intravenous injections over 14 days. The mice were sacrificed on day 14. (e) Western blot analysis of Pgp expression levels in excised MCF-7/ADR tumor tissues treated with PBS, BCNC-3, free Dox, siNC/Dox-BCNC-3, and Pgp-siRNA/Dox-BCNC-3. Quantitative Pgp expression levels were calculated by normalizing band intensities to β-actin and subsequently dividing by the intensity of the PBS group. (f) Histological analysis of excised MCF-7/ADR tumors treated with PBS, siNC-BCNC-3, free Dox, and Pgp-siRNA/Dox-BCNC-3 using hematoxylin and eosin staining and TUNEL assay. Tumor cell nuclei were stained blue, and apoptotic tumor cells were stained brown. Data are presented as the mean ± standard deviation (SD) (*n* = 5). The significance values were obtained by comparing the study trials to the PBS group. *P < 0.05.

Specifically, to test the stability of Dir-BCNC-3 in physiologically mimicking media, Dir release from Dir-BCNC-3 in a pH 7.4 PBS solution was performed. Dir selectively exhibits fluorescence in a hydrophobic environment, such as the inner cavity, while having very weak fluorescence in an aqueous medium.^98^ Correspondingly, the release of Dir from Dir-BCNC-3 can result in a decrease in the original fluorescence emission intensity. By measurement of the decreased fluorescence intensity of Dir-BCNC-3, the cumulative Dir release from Dir-BCNC-3 can thus be evaluated. According to the fluorescence emission spectra at different incubation times (Figure 7c), the Dir release profile of Dir-BCNC-3 at pH 7.4 was plotted (Figure 7d). According to the release result, Dir-BCNC-3 retained 75.1 and 71.7% of the loaded Dir after 48 and 72 h incubation, respectively, indicating that Dir-BCNC-3 possesses good colloidal stability and can preserve the fluorescence activity of Dir. This carrier property makes Dir-BCNC-3 a suitable fluorescence probe for evaluating the biodistribution of BCNC-3.

Moreover, due to concern about the cardiotoxicity of Dox, the serum biochemicals of mice were analyzed after 14 days of intravenous injection with either free Dox or Dox-BCNC-3, at a Dox concentration of 4 mg/kg based on the weight of each test mouse.^99^ These biochemicals included creatine kinase (CK), lactate dehydrogenase (LDH), and serum creatine kinase-MB (CK-MB) (Figure 8). Among these, CK and LDH are the major enzymes to evaluate myocardial ischemia necrosis, while CK-MB serves as a marker to detect a heart attack.^99,100^ The elevation of these values indicates an increased risk of heart attack. Compared with the PBS group, the free Dox group showed a significant increase in all three measured biochemicals, indicating that free Dox severely affected the heart and damaged myocardial cells (Figure 8). However, relative to the free Dox group, the Dox-BCNC-3 group exhibited considerably reduced levels of CK, LDH, and CK-MB, suggesting that encapsulation with BCNC-3 can mitigate the cardiotoxicity of Dox.

The antitumor efficacy of BCNC-3-mediated drug formulations was preliminarily evaluated in MCF-7/ADR xenograft tumor-bearing mice (Figure 9). As for the PBS group, no significant change in the body weight of the test mice was observed from the BCNC-3-treated groups with or without loading of therapeutics (Figure 9a). In contrast, the free Dox group experienced a 6.5% weight loss relative to the initial weight after 14 days, reflecting the toxic effect of free Dox. Compared with the PBS group, the BCNC-3 group exhibited a similar tumor volume (Figure 9b). Meanwhile, the tumor volumes following treatment with the various therapeutic formulations followed the order: free Dox > siNC/Dox-BCNC-3 > Pgp-siRNA/Dox-BCNC-3. At the end of the experiment, the ex vivo tumor volumes and weights were also measured (Figure 9c,d). The antitumor efficacy of the test groups from those results was in accordance with that evaluated from observing the tumor volume change. Such results indicate that the antitumor efficacy of Dox can be increased via the encapsulation of BCNC-3. This is because BCNC-3 enables the encapsulated Dox to have a prolonged blood circulation time and to decrease the blood clearance, subsequently resulting in the EPR effect on the treated tumor.^93^ With the loading of Pgp-siRNA, it was considered that Pgp-siRNA/Dox-BCNC-3 successfully sensitized the tumor cells through inhibiting the Pgp expression, thus exhibiting the highest antitumor efficacy among the test groups.^93^

To demonstrate the Pgp silencing effect of Pgp-siRNA/Dox-BCNC-3 on the MCF-7/ADR xenograft tumor tissues, Western blot analysis has been conducted on the excised tumor tissues from the PBS, free Dox, and BCNC-3 relevant groups (Figure 9e). Using the band intensity from the PBS group as the reference, the Pgp expression level from the BCNC-3, free Dox, and siNC/Dox-BCNC-3 groups was measured to range from 96.0 to 78.1%, implying they had no significant Pgp silencing effect.^91,101^ Meanwhile, the Pgp-siRNA/Dox-BCNC-3 group showed a Pgp expression level of 38.0%, indicating that the delivery of Pgp-siRNA via BCNC-3 can downregulate the Pgp expression from the xenograft tumor tissues. Such a result is consistent with the in vitro Pgp silencing result of Pgp-siRNA/BCNC-3 on MCF-7/ADR cells (Figure 5b), suggesting the acceptable Pgp-siRNA delivery efficiency of BCNC-3. However, optimizing the in vivo siRNA delivery efficiency is still required through tuning their surface properties and particular structures.

The excised tumor tissues from the PBS, siNC-BCNC-3, free Dox, and Pgp-siRNA/Dox-BCNC-3 groups have been further treated with H&E staining and analyzed via a terminal deoxynucleotidyl transferase dUTP nick end labeling (TUNEL) assay to evaluate and compare their antitumor efficacy (Figure 9f). H&E results showed that most excised tumor tissue from the PBS and siNC-BCNC-3 groups had a high nuclear-cytoplasmic ratio.^102^ This ratio was observed to decrease with free Dox and was further considerably reduced with Pgp-siRNA/Dox-BCNC-3, indicating that Pgp-siRNA/Dox-BCNC-3 had significantly improved antitumor efficacy compared with free Dox and the PBS group. The TUNEL assay, which can detect DNA fragmentation in tumor cell nuclei, allows staining of necrotic tumor cells and subsequent quantification of apoptotic cells.^103^ Corroborating the H&E results, TUNEL assay revealed that the PBS group exhibited an apoptotic cell level below 10%, whereas siNC-BCNC-3, free Dox, and Pgp-siRNA/Dox-BCNC-3 showed levels of 14.7, 47.4, and 79.6%, respectively. These findings suggest that the antitumor activity of Dox can be improved by Pgp-siRNA/Dox-BCNC-3 through its carrier functions and the Pgp silencing effect. Moreover, BCNC-3-based siRNA complexes have no significant concern on the nonspecific immune stimulation to tumor tissues because of the negligible apoptotic cell level from siNC-BCNC-3.

In addition, major organs, such as the liver, kidneys, spleen, lungs, and heart, were harvested from Pgp-siRNA/Dox-BCNC-3-treated mice after 14 days of treatment and analyzed histologically via H&E staining (Figure S17). Compared with the PBS group, the Pgp-siRNA/Dox-BCNC-3 group showed no significant morphological changes or pathological abnormalities in any of the examined organs. These histological results indicate that the Pgp-siRNA/Dox-BCNC-3 formulation effectively inhibits tumor growth without causing notable organ toxicity. Optimization of the Pgp-siRNA/Dox-BCNC-3 formulation for in vivo antitumor efficacy and biosafety is currently ongoing in our laboratory.

Overall, it has been demonstrated that BCNC-3 is able to moderately increase the antitumor efficacy of the loaded Dox via the evading effect in MCF-7 and MCF-7/ADR cells. Moreover, with the load of Pgp-siRNA, Pgp-siRNA/Dox-BCNC-3 can significantly improve the antitumor efficacy of Dox compared with Dox-BCNC-3, through Pgp silencing in MCF-7/ADR and MES-SA/Dx5 cells. Preliminary in vivo studies demonstrated that BCNC-3 decreases the cardiotoxicity of Dox while increasing its antitumor efficacy in MCF-7/ADR-bearing mice. These results suggest that BCNC-3 is a promising platform to enhance the antitumor efficiency of various hydrophobic anticancer drugs against different MDR cancer cells through optimal drug/gene combination and its drug loading and delivery capabilities.

## Conclusions

In this study, a new triblock copolymer, PEG-PLA-CPLAs, was successfully synthesized via ROP and thiol–ene modification followed by thorough characterization of its chemical structure. Owing to its amphiphilic properties, PEG-PLA-CPLAs were converted into BCNCs with controllable particle sizes using our developed nanoemulsion interfacial cross-linking method. One optimized formulation, BCNC-3, exhibited tunable charge and surface PEG densities, their ability to coload hydrophobic Dox and hydrophilic Pgp-siRNA, and a sequential release profile for the loaded therapeutics. In addition, BCNC-3 demonstrated improved colloidal stability in a physiologically mimicking environment due to the incorporated cross-linkages. As a carrier for Dox, BCNC-3 significantly enhanced cellular uptake in MCF-7/ADR cells, a MDR cancer cell line, via an evasion effect. As a vector for Pgp-siRNA, BCNC-3 enabled effective Pgp silencing and facilitated endosomal and lysosomal escape in MCF-7/ADR cells. When coloaded with these two chemically distinct therapeutics, the resulting Pgp-siRNA/Dox BCNCs exhibited superior antitumor efficacy against MCF-7/ADR cells compared with other control groups, both in vitro and in vivo. In addition, Pgp-siRNA/Dox BCNCs have demonstrated Pgp silencing and antitumor effects in other MDR cancer cells such as MES-SA/Dx5 cells. Owing to their carrier functions, BCNCs are considered a promising platform for a wide range of drug/gene combination therapies, with potential applications in treating various intractable diseases beyond cancer.

## Supplementary Material



## Notes

Details for animal experiments and tumor implantation procedures; protocols for in vivo biodistribution study, antitumor efficacy evaluation and toxicity test; chemical structure of CPLA polymers (Figure S1); appearances of prepared nanoemulsions after ultrasonication treatment (Figure S2); TEM images of Dox-BCNC-3 and Pgp-siRNA/Dox-BCNC-3 (Figure S3); DLS analyses of non-cross-linked and cross-linked BCNC formulations under physiologically relevant conditions (Figures S4–S6); GPC degradation profiles of PEG-PLA-CPLA-80 and CPLA-60 at different pH values (Figure S7); RNase protection and pH-dependent siRNA release behaviors of Pgp-siRNA-BCNC-3 (Figures S8–S10); cumulative release profiles of Dox and Pgp-siRNA from Pgp-siRNA/Dox-BCNC-3 (Figure S11); in vitro cytotoxicity, cellular uptake, and colocalization analyses in multiple cell lines (Figures S12, S13, S15, and S16); Western blot quantification of Pgp expression in MES-SA/Dx5 cells (Figure S14); histological examination of major organs from treated mice (Figure S17); and synthesis, structural characterization, and physicochemical properties of PEG-PLA-APLA terpolymers and BCNC formulations (Tables S1 and S2) (PDF)
