# Supplementary material for: Chemically Cross-Linked Nanocapsules from Terpolymers of Polyethylene Glycol-Polylactide with Cationic Pendent Groups for Pgp-siRNA and Doxorubicin Codelivery in Overcoming Multidrug Resistance in Breast Cancer
Source: Mol Pharm. 2025 Nov 28;23(1):79–102. doi: 10.1021/acs.molpharmaceut.4c00600 (PMC12776577; doi:10.1021/acs.molpharmaceut.4c00600)
Supplement: Supplementary file 1 [file mp4c00600_si_001.pdf]

Supporting Information

Chemically cross-linked nanocapsules from terpolymers of polyethylene glycol-polylactide with cationic pendent groups for P-gp siRNA and doxorubicin co-delivery in overcoming multidrug resistance in breast cancer

Zheng-Ian Lin [a,b,1], *Tzu-Hsien Tsai* [c,1], *Li-Hsien Wu* [d], *Chien-Hou Lu* [e], *Chih-Yuan Hsu* [a], *Haiqing Liang* [f], *Hsin-Yu Lin* [a], *Yi-Ping Fang* [h,i,j], *Wing-Cheung Law* [k], *Cheng-Hsi Chang* [c], *Hau-En Liou* [c], *Chengbin Yang* [f,g]*, *Pei-Wen Cheng* [b,l]*, *Che-Hsin Lee* [d,m,n,o,p]*, *Chih-Kuang Chen* [a]*

[a] Polymeric Biomaterials Laboratory, Department of Materials and Optoelectronic Science, National Sun Yat-Sen University, Kaohsiung 80424, Taiwan

[b] Department of Medical Education and Research, Kaohsiung Veterans General Hospital Kaohsiung 81362, Taiwan

[c] Division of Cardiology and Department of Internal Medicine, Ditmanson Medical Foundation Chiayi Christian Hospital, Chiayi 60002, Taiwan

[d] Department of Biological Sciences, National Sun Yat-sen University, Kaohsiung 80424, Taiwan.

[e] Department of Fiber and Composite Materials, Feng Chia University, Taichung 40724, Taiwan

[f] Guangdong Key Laboratory for Biomedical Measurements and Ultrasound Imaging, School of Biomedical Engineering, Shenzhen University Medical School, Shenzhen University, Shenzhen, Guangdong 518060, P. R. China

[g] The Shenzhen Key Laboratory of Metabolism and Cardiovascular Homeostasis, Shenzhen University, Shenzhen 518060, China

[h] School of Pharmacy, College of Pharmacy, Kaohsiung Medical University, Kaohsiung 80708, Taiwan

[i] Department of Medical Research, Kaohsiung Medical University Hospital, Kaohsiung 80708, Taiwan

[j] Regenerative Medical and Cell Therapy Center, Kaohsiung Medical University, Kaohsiung 80708, Taiwan

[k] Department of Industrial and Systems Engineering, The Hong Kong Polytechnic University, Hung Hom, Hong Kong, 999077 P. R. China

[l] Department of Biomedical Sciences, National Sun Yat-Sen University, Kaohsiung 80424, Taiwan

[m] Aerosol Science Research Center, National Sun Yat-sen University, Kaohsiung 80424, Taiwan.

[n] College of semiconductor and advanced technology Research, National Sun Yat-sen University, Kaohsiung 80424, Taiwan.

[o] Department of Medical Laboratory Science and Biotechnology, Kaohsiung Medical University, Kaohsiung 80708, Taiwan.

[p] Department of Medical Research, China Medical University Hospital, China Medical University, Taichung 404327, Taiwan.

[1] These authors contributed equally to this work.

SUPPORTING EXPERIMENTAL METHODS

In vivo biodistribution. When the tumor volume reached approximately 150 mm^3, tumor-bearing mice (n = 3) were intravenously administered 100 μL of free 1,1-dioctadecyl-3,3,3,3-tetramethylindotricarbocyanine iodide (Dir) and Dir-loaded BCNC-3 (Dir-BCNC-3) at a Dir concentration of 0.625 mg/kg (calculated based on the body weights of the test mice) using a pH = 7.4 PBS solution as the diluting medium. The formulation of Dir-BCNC-3 was the same to that of Dox-BCNC-3, except for the loaded agent. Fluorescence imaging of the mice was performed at 2, 12, 24, 48, and 72 h post-injection using an IVIS spectrum optical imaging system (excitation, 750 nm; emission, 780 nm; PerkinElmer, Inc.) to acquire whole-body fluorescence images. After the final imaging, animals were sacrificed, and major organs were collected and imaged by the IVIS spectrum optical imaging system. The radiance (photon emission per unit area) of a regions of interest (ROI) was acquired by the Living image software.

In vivo antitumor efficacy. When the tumor volume has reached up to approximately 150 mm^3, MCF-7/ADR tumor-bearing mice were randomly divided into five groups (n = 5 per group). The mice were treated via intravenous injection with a pH = 7.4 PBS solution, BCNC-3, free DOX, siNC/Dox-BCNC-3, or Pgp-siRNA/Dox-BCNC-3 every two days, resulting in a total injection time of six. For each injection, the dose amounts for siRNA and Dox were set at 40 μg and 68.4 μg. Tumor volumes and body weights were measured every other day throughout the treatment period. Tumor volume (V, mm^3) was calculated using the formula: V

(mm^3) = (length (mm) × width^2)/2. At the end of the observational period, the mice were sacrificed, followed by harvesting the tumor tissues for imaging records. At the end of the observational period, the mice were sacrificed, and the major organs (e.g., liver, kidneys, spleen, lungs and heart) from the PBS, Free Dox and Pgp-siRNA/Dox-BCNC-3 groups were harvested for histological analysis upon hematoxylin and eosin (H&E) staining. To assess apoptotic activity within tumor tissues, frozen tumor sections harvested on day 14 were subjected to a terminal deoxynucleotidyl transferase-mediated dUTP nick-end labeling (TUNEL) assay and H&E staining. Both of the staining and microscopic observation were operated and analyzed through the assistance of SIDSCO Biomedical Co., Ltd..

In vivo toxicity study. To evaluate the biosafety of Dox-BCNC-3, a Dox-BCNC-3 solution and a free Dox solution were introduced into mice via intravenous injection at a Dox concentration of 4 mg/kg based on the weight of the test mice. The injection was conducted every two days, resulting in a total injection time of six during the experimental period. The treated mice were sacrificed on day 14 post-injection, and the blood samples were collected for serum analysis. Creatine kinase (CK), creatine kinase isoenzymes (CK-MB), and lactate dehydrogenase (LDH) levels were measured using a Beckman DxC 700 AU automatic biochemical analyzer (Beckman Coulter, Inc.).

SUPPORTING FIGURES

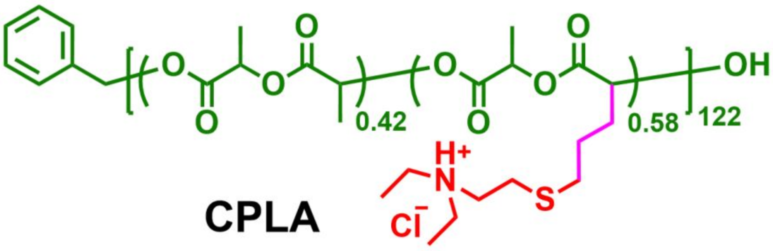

Figure S1. Chemical structure of CPLA-60, one of the CPLA polymers.

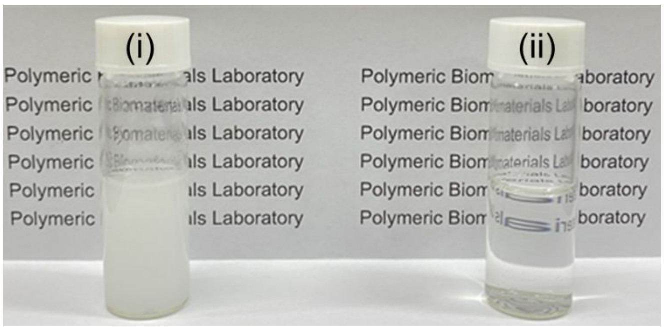

Figure S2. Appearances of a nano-emulsion consisting of 6 mg of PEG-PLA-CPLA-80, 110 μL of chloroform and 4 mL of an aqueous solution containing 0.05 wt% of I2959 after (i) 10 min ultrasonication and (ii) 30 min ultrasonication treatments.

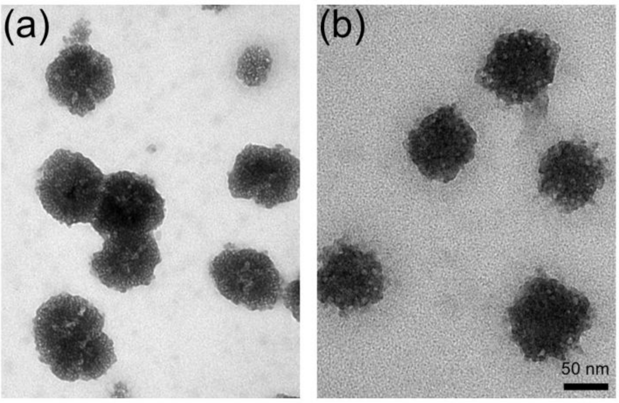

Figure S3. TEM images of (a) Dox-BCNC-3 and (b) Pgp-siRNA/Dox-BCNC-3.

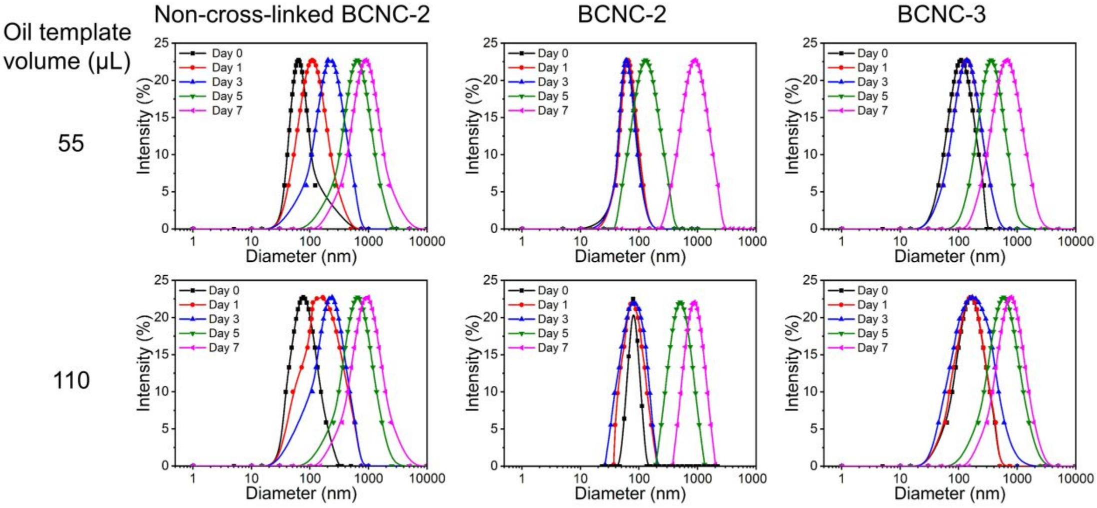

Figure S4. DLS curves of non-cross-linked BCNC-2, BCNC-2 and BCNC-3 with different oil template amounts after incubated in a pH = 7.4 PBS solution at 37 ^{o}C for 1, 3, 5 and 7 days.

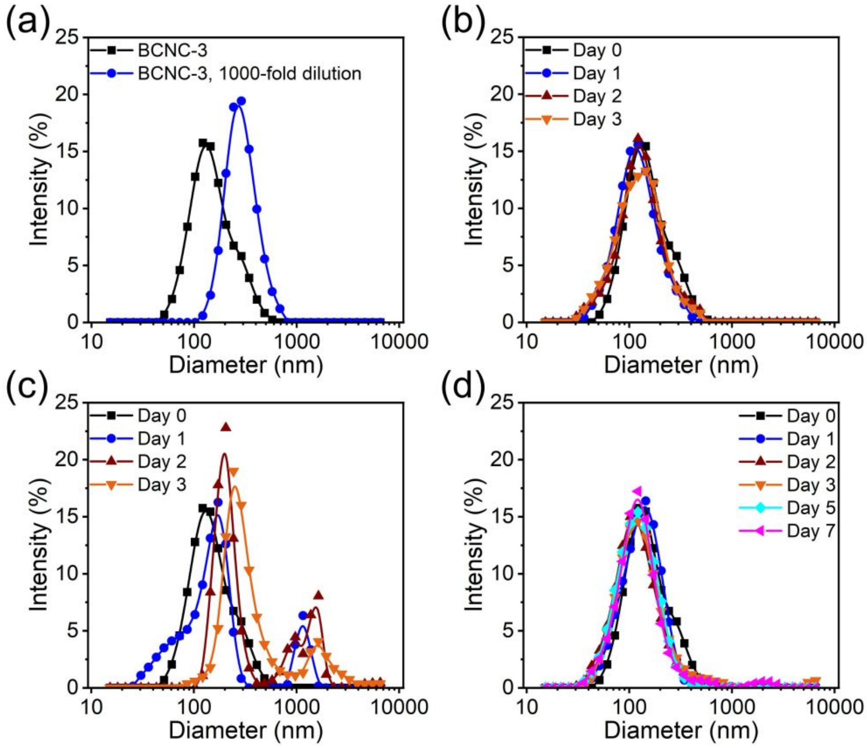

Figure S5. DLS results of BCNC-3 after being incubated at different physiologically-mimicking conditions at predetermined time intervals. The conditions were shown as follows. (a) 1000-fold dilution; (b) 0.1 M NaCl solution at 37 °C; (c) esterase solution (1 mg/mL) at 37 °C and (d) de-ionized water at 25 °C. The particle size of BCNC-3 was measured for three times under the specified condition. One representative result among the measurements was selected to be plotted to the corresponding DLS curve.

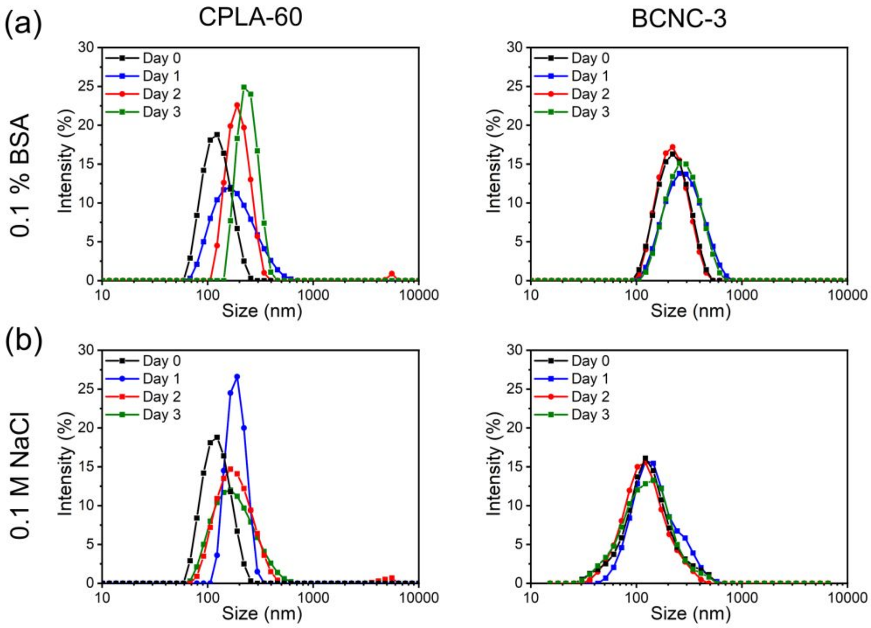

Figure S6. DLS analysis of CPLA-60 and BCNC-3 after incubation under physiologically relevant conditions at predetermined time intervals. The experimental conditions were shown as follows: (a) 0.1% BSA solution (1 mg/mL) and (b) 0.1 M NaCl solution at 37 °C. The particle size of CPLA-60 and BCNC-3 was measured three times under each condition. A representative measurement was selected and plotted in the corresponding DLS curve.

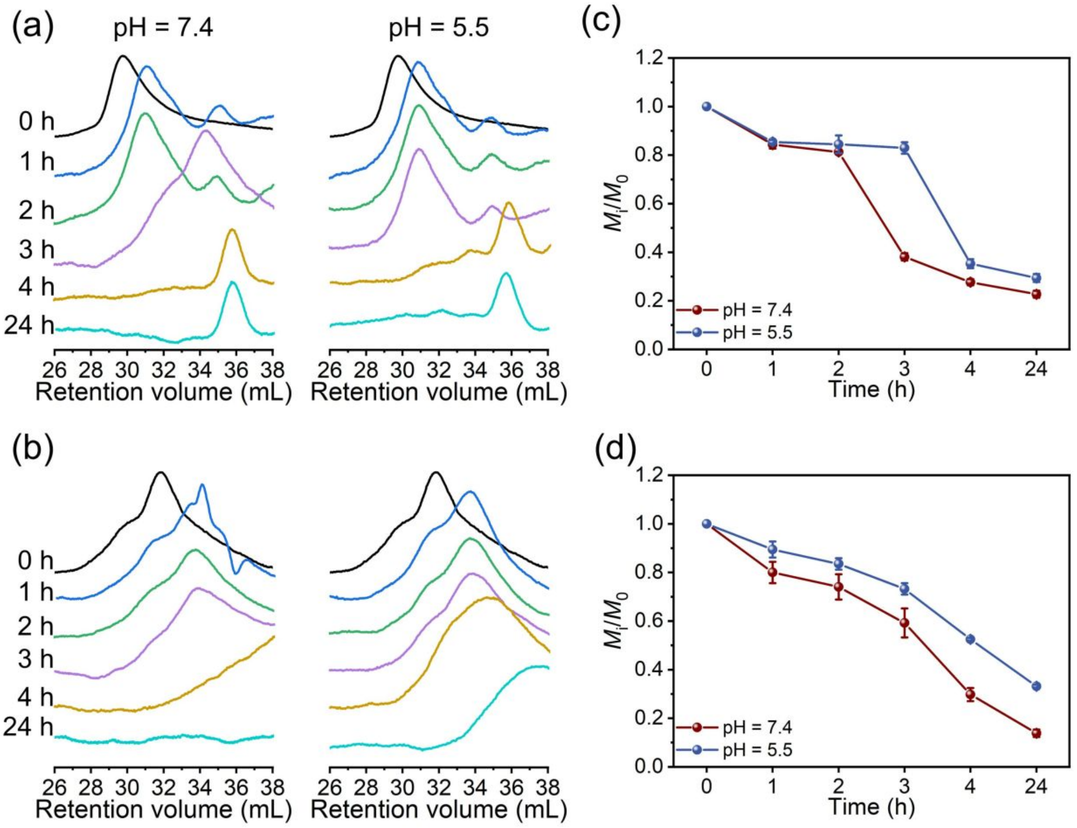

Figure S7. GPC curves of (a) PEG-PLA-CPLA-80 and (b) CPLA-60 after being incubated at pH =7.4 and pH =5.5 for predetermined incubation times. M_i/M_0 values of (c) PEG-PLA-CPLA-80 and (d) CPLA-60 resulting from the corresponding GPC results, in which the repeating time was two (n = 2).

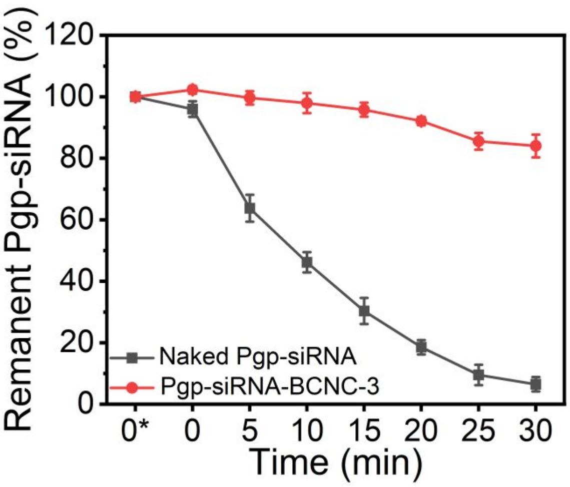

Figure S8. Residual siRNA percentages from naked Pgp-siRNA and Pgp-siRNA-BCNC-3 after incubation in a RNase-containing PBS solution (pH = 7.4) for 5-30 min at a RNase concentration of 0.5 U (10 μg) by using the brightness of intact siRNA as the reference and ImageJ as the processing software.

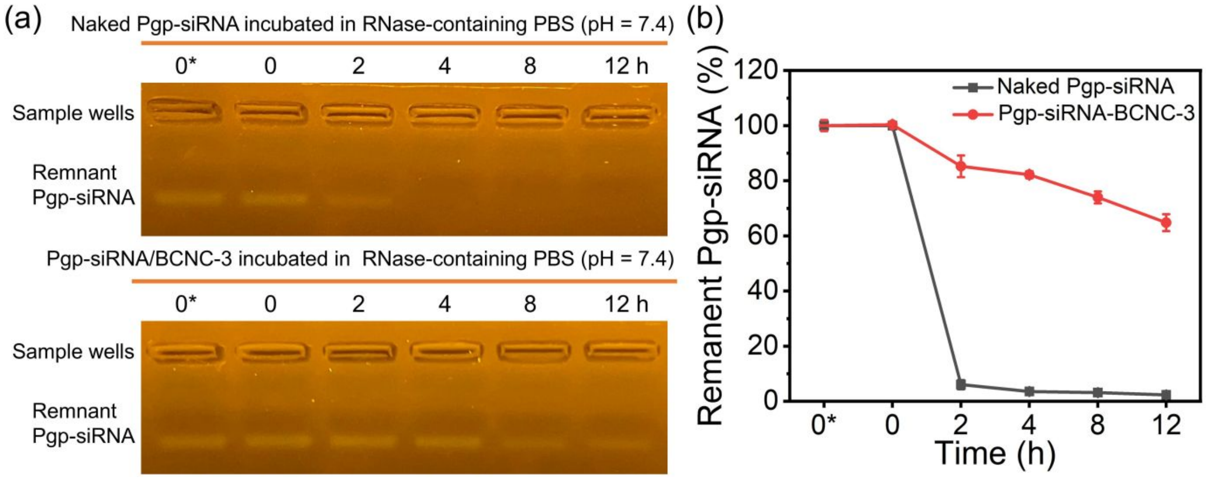

Figure S9. Agarose gel electrophoresis results of (a) naked Pgp-siRNA and Pgp-siRNA-BCNC-3 after 2-12 h incubation in a RNase-containing PBS solution (pH = 7.4). Following the incubation, the siRNA molecules were detached by SDS prior to electrophoresis. The lane labeled "0*" corresponds to intact siRNA without incubating in a RNase-containing solution; (b) residual siRNA percentages of naked Pgp-siRNA and Pgp-siRNA-BCNC-3 after 2-12 h incubation in a RNase-containing PBS solution (pH = 7.4) at a RNase concentration of 0.5 U (10 μg). The residual percentage was determined by comparing the band intensities of test samples to that of the intact siRNA via Image J.

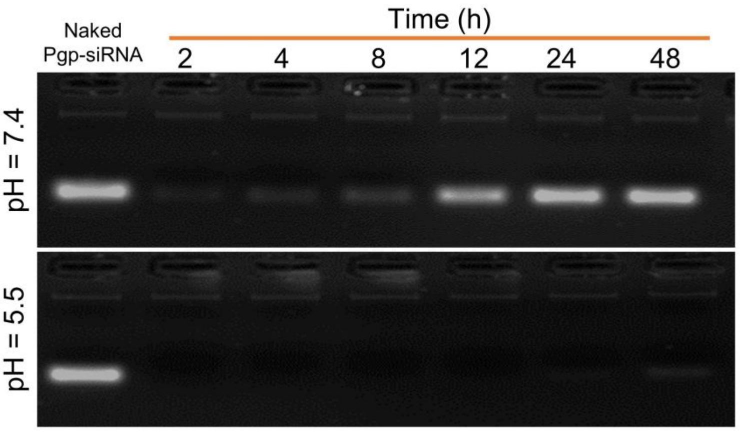

Figure S10. Residual Agarose gel electrophoresis results of Pgp-siRNA-BCNC-3 incubating in pH = 7.4 and pH = 5.5 PBS solutions for 2-48 h. The Pgp-siRNA release percentages of Pgp-siRNA-BCNC-3 were calculated through dividing the band brightness at each release time point by the band brightness of naked Pgp-siRNA, which is considered the intensity of the fully released siRNA from Pgp-siRNA-BCNC-3.

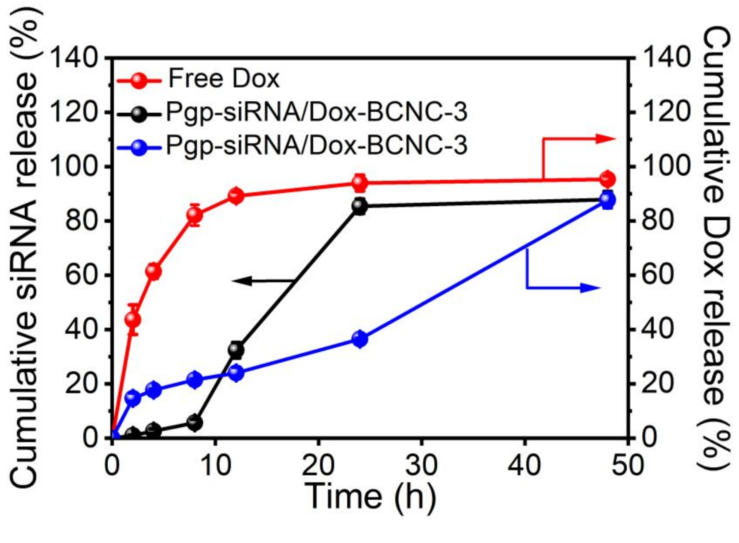

Figure S11. Comparison of the release profiles among free Dox and Dox/Pgp-siRNA from Pgp-siRNA/Dox-BCNC-3 in a pH = 7.4 PBS solution.

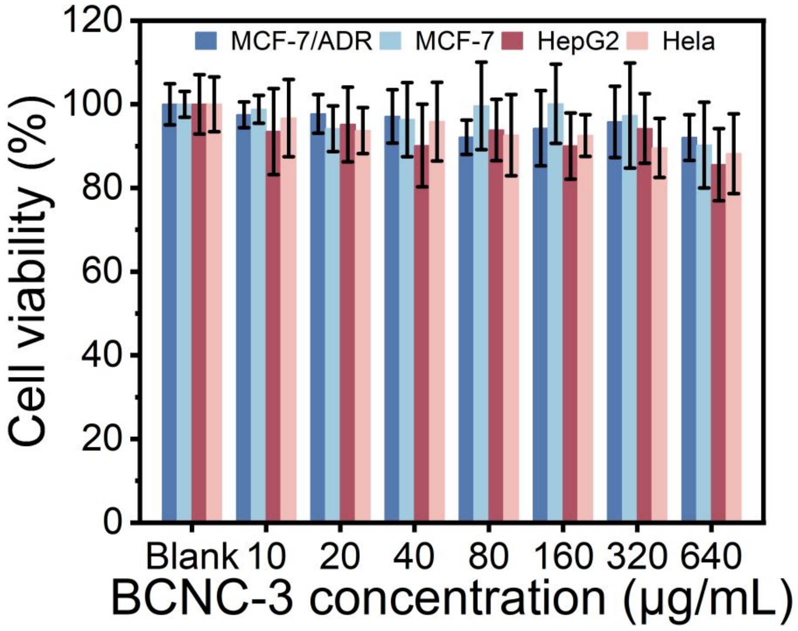

Figure S12. Cell viability results of BCNC-3 at the cell lines of MCF-7/ADR, MCF-7, HepG2 and Hela cells after incubation for 48 h through a MTT assay.

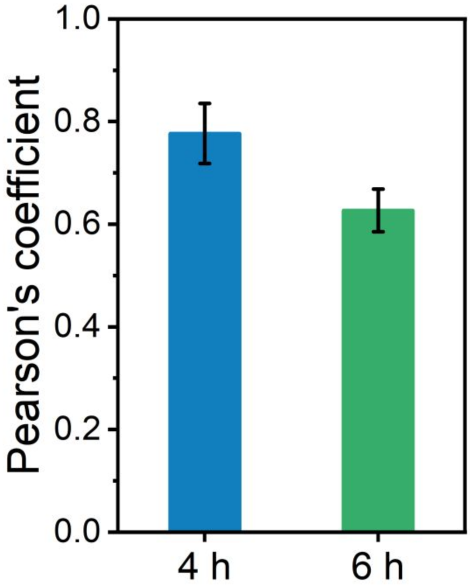

Figure S13. Pearson's coefficients of siFAM/Lyso-Tracker Red in MCF-7/ADR cells after 4 h and 6 h of incubation.

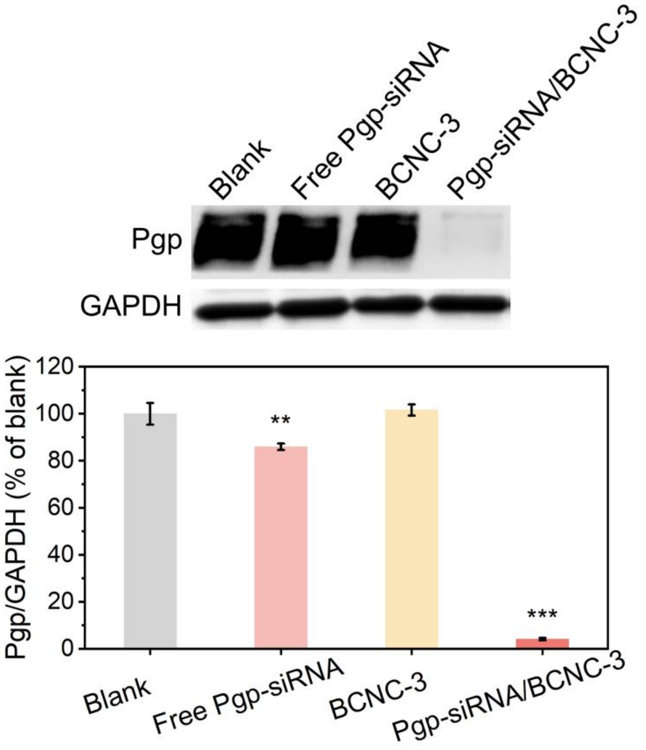

Figure S14. Western blot analysis of Pgp expression levels in MES-SA/Dx5 cells treated with nothing (blank), free Pgp-siRNA, BCNC-3 and Pgp-siRNA/BCNC-3 for 48 h. Using the band darkness from GAPDH as the reference, quantitative Pgp expression levels were obtained by dividing the band darkness intensities from the trials to that from blank. The significance values were obtained by comparing the study trials to the blank group. *$P < 0.05$; **$P < 0.01$; ***$P < 0.001$.

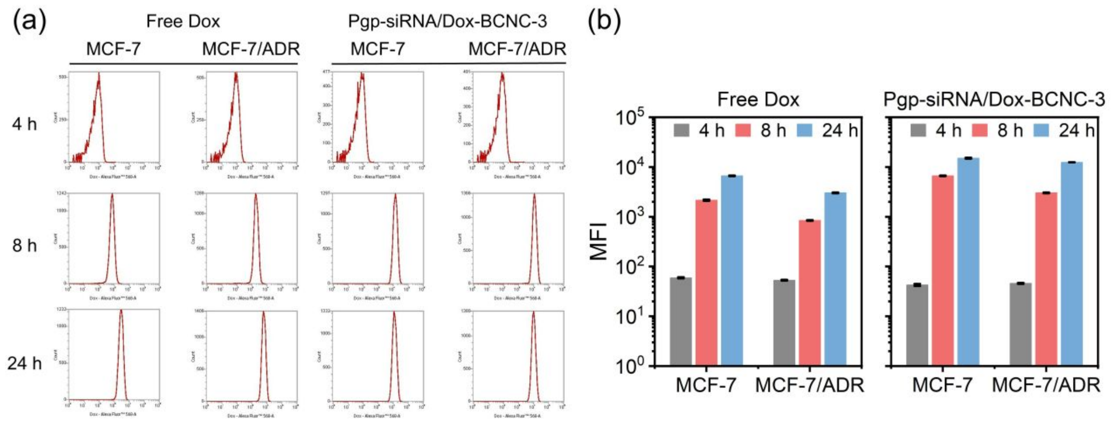

Figure S15. (a) Cellular uptake and (b) quantitative mean fluorescence intensity (MFI) analysis of free Dox and Pgp-siRNA/Dox-BCNC-3 in MCF-7 and MCF-7/ADR cells from flow cytometry measurements after 4, 8 and 24 h incubation.

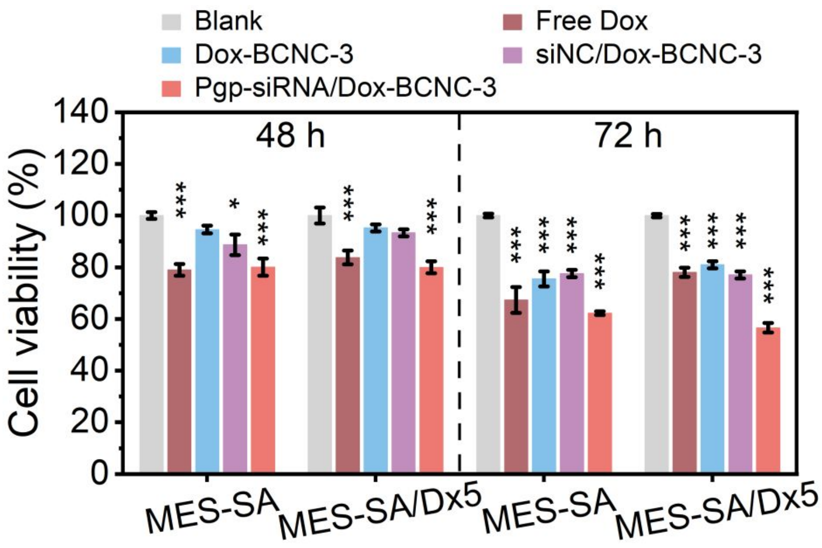

Figure S16. Cell viability of MES-SA and MES-SA/Dx-5 cells treated by free Dox, Dox-BCNC-3, siNC/Dox-BCNC-3 and Pgp-siRNA/Dox-BCNC-3 after 48 h and 72 h incubation through a MTT assay, in which the concentrations of Pgp-siRNA/siNC siRNA and Dox were 0.4 and 0.5 μg/mL.Error bars represent standard deviation values resulting from three independent experiments. The significance values were obtained by comparing the study trials to the blank group. *$P < 0.05$; **$P < 0.01$; ***$P < 0.001$.

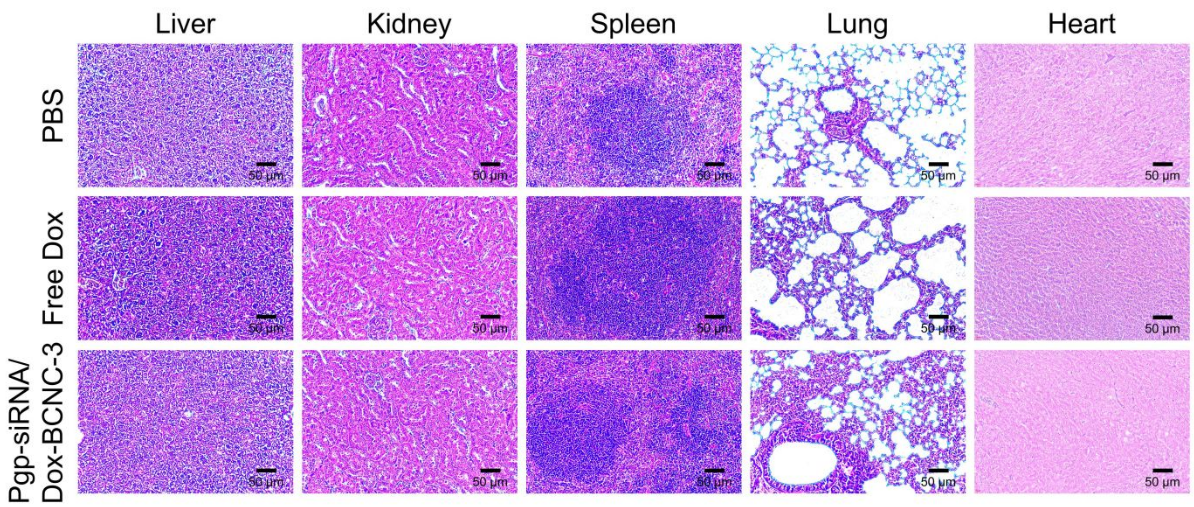

Figure S17. H&E staining results of major organs harvested from the mice treated with PBS, free Dox, and Pgp-siRNA/Dox-BCNC-3 after 14 days of treatment.

SUPPORTING TABLES

Table S1. Synthesis and characterization of PEG-PLA-APLAs as the precursors of PEG-PLA-CPLAs through a sequential ROP procedure.

Entry[a)]	$[\text{initiator}]_0:[\text{ALA}]_0:[\text{LA}]_{72}$[b)]	M_n^{NMR} (kDa)[c)]	M_n^{GPC} (kDa)[d)]	PDI[d)]
PEG_{45}-*b*-PLA_{56}-*b*-$APLA_{42}$	1: 60: 60	17.2	12.1	1.33

a) The subscript numbers imply the DP value for the corresponding repeating unit; *b)*The subscript numbers indicate the time (unit: hr) at which the corresponding monomer was added into the ROP system; *c)* Calculated from ^{1}H NMR; *d)* Relative to linear polystyrenes and dimethylformamide (DMF) as the effluent solvent.

Table S2. Particulate characterization of BCNC-3, Dox-BCNC-3 and Pgp-siRNA/DOX-BCNC3.

Entry	$D_{h,i}$ (nm)/ PDI	Zeta potential (mV)
BCNC-3 [a)]	118 ± 5.2 / 0.158 ± 0.02	35.7 ± 1.1
Dox-BCNC-3 [a)]	133 ± 4.1/ 0.216 ± 0.05	35.8 ± 0.7
Pgp-siRNA/Dox-BCNC-3 [a)]	138 ± 1.3 / 0.231 ± 0.03	28.7 ± 0.8

a) The $D_{h,i}$ and the zeta potential of the BCNCs were measured for five times by DLS to obtained the average values, and the error bars were calculated from five independent experiments.
